# The benefits and harms of oral iron supplementation in non-anaemic pregnant women: a systematic review and meta-analysis

**DOI:** 10.1093/fampra/cmae079

**Published:** 2025-01-21

**Authors:** Archie Watt, Holden Eaton, Kate Eastwick-Jones, Elizabeth T Thomas, Annette Plüddemann

**Affiliations:** Oxford Medical School, Medical Sciences Division Office, Level 3 Academic Centre, John Radcliffe Hospital, Headington, Oxford OX3 9DU, United Kingdom; Oxford Medical School, Medical Sciences Division Office, Level 3 Academic Centre, John Radcliffe Hospital, Headington, Oxford OX3 9DU, United Kingdom; Oxford Medical School, Medical Sciences Division Office, Level 3 Academic Centre, John Radcliffe Hospital, Headington, Oxford OX3 9DU, United Kingdom; Nuffield Department of Primary Care Health Sciences, Centre for Evidence Based Medicine, University of Oxford, Radcliffe Primary Care Building, Radcliffe Observatory Quarter, Woodstock Road, Oxford OX2 6GG, United Kingdom; Nuffield Department of Primary Care Health Sciences, Centre for Evidence Based Medicine, University of Oxford, Radcliffe Primary Care Building, Radcliffe Observatory Quarter, Woodstock Road, Oxford OX2 6GG, United Kingdom

**Keywords:** anaemia, iron deficiencies, iron, pregnancy, care, prenatal

## Abstract

**Background:**

Iron deficiency during pregnancy poses a significant risk to both maternal and foetal health. Current international guidelines provide discrepant advice on antenatal iron supplementation for non-anaemic women.

**Objective:**

We aimed to quantify the benefits and harms of routine antenatal supplementation in non-anaemic women.

**Methods:**

The Cochrane Library, MEDLINE, Embase, and clinical trial registries were searched for randomized controlled trials and observational studies comparing oral iron supplementation with placebo or no supplement in non-anaemic pregnant women. Risk of bias was assessed for each study and the results were synthesized via meta-analysis.

**Results:**

Twenty-three eligible studies were identified with 4492 non-anaemic pregnant women. Supplemented groups had higher haemoglobin [mean difference = 6.95 g/l, 95% confidence interval (CI): 4.81–9.09, *P* < .001, moderate certainty, *I*^2^ = 91%] and ferritin (mean difference = 12.22 ng/ml, 95% CI: 6.92–17.52, *P* < .001, moderate certainty, *I*^2^ = 87%) and were at lower risk of anaemia (relative risk = 0.50, 95% CI: 0.34–0.74, *P* < .001, high certainty, *I*^2^ = 42%, number needed to treat (NNT) = 10). There was no difference in birth weight, preterm birth, and rate of caesarean section. Reporting on harms was inconsistent and there was insufficient evidence to determine an association between iron supplements and any negative outcome.

**Discussion:**

Prophylactic iron supplementation likely results in a large reduction in maternal anaemia during pregnancy. Future research should qualify the impact of this benefit on women’s quality of life and determine which subpopulations benefit most. Evidence surrounding the harms of iron supplementation in the non-anaemic population is poor quality and inconsistent. Randomized controlled trials quantifying the risk of gastrointestinal (GI) disturbance and iron overload are essential to inform iron supplement use and reduce unwarranted variations in international guidelines.

**Key messages**

- International guidelines on antenatal iron supplements for non-anaemic women vary.
- We found iron supplements are likely to lower the risk of anaemia.
- Evidence on the harms of iron supplements in this population is currently inconsistent.
- Future research should quantify harms and determine the impact on quality of life.

## Background

Iron requirements increase during pregnancy as a result of an expanding red blood cell mass. Without sufficient iron intake, iron reserves are depleted, red blood cell production is impaired and iron deficiency anaemia (IDA) can develop. IDA affects up to 36.5% of pregnant women globally [1, 2], making it the most common nutritional disorder worldwide. IDA poses substantial risks to both maternal health and foetal health and development, increasing the risk of premature birth, postpartum depression, and low infant birth weight, according to a meta-analysis of 117 studies globally [3]. There is also emerging evidence of maternal and foetal harms even before iron deficiency (ID) progresses to IDA [4]. Infants born of iron-deficient mothers are themselves at increased risk of ID, and may develop impaired cognitive, motor, and social–emotional function [5, 6].

As a preventative measure, the World Health Organisation (WHO) and other international guidelines advise that all pregnant women, regardless of iron status, receive iron supplementation alongside folic acid [7–10]. In contrast, guidelines from other countries, including the UK, USA, and Australia, either recommend against or fail to address universal supplementation, highlighting clinical uncertainty with regard to its benefits for pregnant women who do not have anaemia [1, 11–13].

Previous reviews have demonstrated significant benefits of iron supplementation, including a 2015 review which found that supplementation reduced maternal anaemia at term by 70% and improved foetal outcomes, such as birth weight [14]. However, this review combined both anaemic and non-anaemic groups in their analysis. The benefits of iron supplementation are already well established for anaemic women, but the extent to which these benefits apply to non-anaemic women is unclear. A 2023 systematic review examined non-anaemic women, but specified that participants must also be iron-replete [15]. While this is clinically applicable to some countries, it is less relevant in countries like the USA, UK, or Australia where iron studies are not included in routine antenatal screening.

Arguments against routine prophylactic iron supplementation in pregnancy suggest that the harms outweigh the benefits for non-anaemic women. Iron overload and high haemoglobin may be associated with adverse outcomes including preterm birth and foetal growth restriction [3, 16, 17]. Oral iron supplements can also cause unwanted gastrointestinal side effects [18], however, these harms have not been clearly quantified in the context of pregnancy.

Given the clinical uncertainty, it is critical to reexamine whether universal iron supplementation would provide a net benefit to this population’s health and wellbeing. Our systematic review explores both the benefits and harms of iron supplementation in non-anaemic pregnant women, a key population in the discussion surrounding routine iron supplementation guidelines globally.

## Methods

The study protocol was registered on OSF (DOI:10.17605/OSF.IO/HKZ4C). This review is reported according to PRISMA reporting standards [19].

Patient encounters in primary care inspired the initial investigation and research into this topic, however, patients and the public were not involved in the undertaking of this study.

### Literature search and extraction

The Cochrane Library, MEDLINE, and Embase (via Ovid) were searched for randomized controlled trials (RCTs) and observational studies published up to 31/01/2024. clinicaltrials.gov and who.int/clinical-trials-registry-platform were searched for additional studies. The search terms used are provided in Supplementary Fig. S1. Reference lists of included papers and systematic reviews were also screened.

We included studies of non-anaemic pregnant women, regardless of iron status, as defined by the studies or NICE guidelines (Hb >110 g/l), taking daily oral iron supplements. Control groups were either placebo or no treatment. Studies were not excluded based on outcome, but predefined outcomes were chosen to reduce selection bias. Primary outcomes were maternal haemoglobin and ferritin, size for gestational age, and whether or not harms were reported. Secondary outcomes were caesarean sections, preterm birth, maternal quality of life, fatigue, gastrointestinal disturbance, gestational diabetes, infections in pregnancy, and any other reported harms. Incidence of anaemia was included as a *post hoc* outcome, since it was frequently reported and is clinically relevant. Birth weight was used instead of ‘size for gestational age’, as this was more commonly reported. RCTs and observational studies were included, however, only RCTs were combined for the meta-analysis. No restriction was placed on publication year or language.

Two reviewers independently screened titles, abstracts and full texts using Rayyan QCRI online software [20] and disagreements were discussed among all reviewers. For trials with multiple publications, data were extracted from the publication with the most comprehensive dataset. Where studies measured outcomes at multiple time points, only the measurements closest to 40 weeks of gestation were used. If studies had multiple intervention or control groups, the means and standard deviations of the groups were combined using the method in Cochrane’s handbook [21]. Data were extracted relating to outcomes, study design, location, number of participants, population characteristics, formulation, and dose. When needed, authors were contacted for additional data. A copy of the data extraction template was published on OSF (DOI:10.17605/OSF.IO/HKZ4C).

### Quality assessment

Risk of bias was independently assessed by two reviewers using the Cochrane risk of bias tool for RCTs [22], or the Newcastle-Ottawa Scale for observational studies [23]. The GRADE approach was used to assess the certainty of the evidence for the primary outcomes [24]. Publication bias was investigated for outcomes with more than 10 trials by examining funnel plots for asymmetry. Alternative reasons were considered for asymmetry, including differences in study methodological quality and true heterogeneity in intervention effects.

### Data synthesis and analysis

Data about benefits were synthesized from RCTs using meta-analysis. Data about harms were synthesized from RCTs and observational studies using narrative review. Statistical analysis was carried out using the meta package in R version 4.0.5 [25]. Trial results were pooled using a random-effects model due to a high degree of methodological heterogeneity. For dichotomous data, results were presented as relative risk (RR) with 95% confidence intervals (CIs). For continuous data, the mean difference (MD) was used. Prediction intervals were presented within the results or in Supplementary Materials.

Heterogeneity was assessed using Chi2, *I*^2^, and tau2 statistics. For outcomes with more than 10 studies, we conducted subgroup analysis based on gestational age at the start of supplementation (first or second trimester), dose (low = <45 mg, high = 45–75 mg, extreme = >75 mg), definition of anaemia used by studies (below, at, or above 110 g/l), and measurement timing (first or second half of the third trimester or at term). To explore clinical heterogeneity, we performed additional subgroup analysis based on human development index (HDI) (low = <0.550, medium = 0.550–0.699, high = 0.700–0.799, very high = >0.799) as data is freely available for each study’s country of origin and year of publication. For statistically significant results, sensitivity analysis was conducted by removing studies determined to be at high risk of bias. *Post hoc* sensitivity analyses were conducted for the anaemia outcome. Publication bias was investigated by removing studies with fewer than 100 participants. The impact of zero count correction was interrogated by reanalysis using Peto’s method [21].

## Results

### Study selection

**Figure 1.**
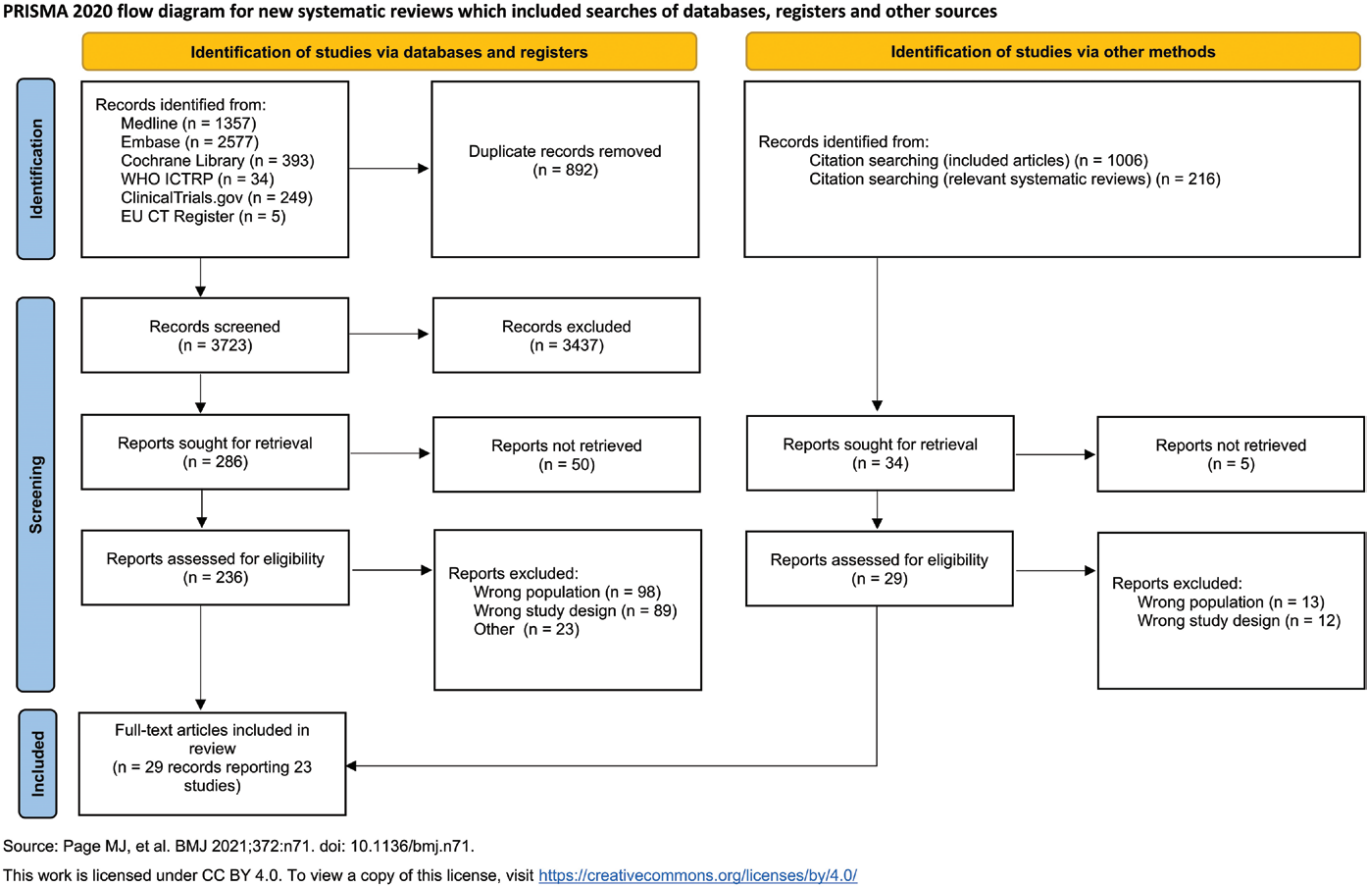
PRISMA study flow diagram.

3723 unique articles were identified and 26 were eligible for inclusion (Fig. 1) [19]. Four articles were identified via reference lists. These 30 eligible articles corresponded to 23 unique studies—21 RCTs and 2 observational studies [26–48].

### Study characteristics

Of 5678 women included at baseline, complete data was available for 4492. Of these, 2296 received iron supplementation, 2050 received a placebo and 155 were non-supplemented controls.

Population characteristics are shown in Table 1. Studies were from a diverse range of countries (six from Iran, four from the USA, two each from Turkey and Denmark, and one each from Sweden, Finland, and Norway, Netherlands, UK, Italy, and Tanzania). Three studies [37, 39, 42] used thresholds for anaemia below 110 g/l and 11 studies also used a ferritin threshold [32–35, 38, 40, 42, 43, 46–48] (see Table 1).

**Table 1.** Table of study characteristics.

Study	Study type	Country	Socio-economic status (if given)	Average age	Number of participants		Definition of anaemia 1 = Hb > 110g/l	Pregnancy		Mean baseline Hb (g/l)		Iron supplementation				Control group
					Intervention	Control		Parity	1 = Singleton 2 = Twin 3 = mixed	Intervention (SD)	Control (SD)	Duration	Formulation	Elemental iron (mg)	Frequency	
Svanberg *et al*. (1975)	RCT	Sweden	-	-	24	26	Hb≥12g/100ml	Primiparous	-	123 (1.8)	125 (1.3)	11- to 14-week gestation to 8- to 10-week postpartum	FeSO_4_	100	BD	Placebo
Puolakka *et al*. (1980)	RCT	Finland	-	26.0	16	15	1	Mixed	1	119 (7)	121 (6)	16-Week gestation to 8-week postpartum	FeSO_4_	200	OD	Non-supplemented
Palgi *et al*. (1981)	Observational	Israel	1.5% upper, 48% middle, 31% lower according to husband’s occupation	-	47	64	1	-	1	118 (6)	121 (1)	16- to 20-week gestation to term	FeSO_4_	100	OD	Non-supplemented
Buytaert *et al*. (1983)	RCT	Netherlands and Belgium	-	-	42	41	Hb>7mmol/l (126g/l)	-	1	^b^R = 128.9 (6.4) A = 127.3 (9.7)	^b^R = 128.9 (8.1) A = 127.3 (8.1)	13-Week (R) OR 16-week (A) gestation to term	FeSO_4_	105	OD	Non-supplemented
Dawson *et al*. (1989)	RCT	USA (Texas)	-	-	20	21	1 ANDhaematocrit≥0.33	-	-	-	-	13-Week gestation to 12-week postpartum	(NI)	18	OD	Placebo
Eskeland *et al*. (1997)	RCT	Norway	1 (iron) vs. 3 (control) low education (elementary school only) 1 (iron) vs. 1 (control) ‘living single’	28.0	24	20	1	Mixed	1	^c^	^c^	13-Week gestation to 24-week postpartum	Ferrous fumarate and heme-iron	27	TD	Placebo
Meier *et al*. (2003)	RCT	USA (Wisconsin)	-	22.6	48	44	1 ANDferritin>12ng/ml	Primiparous	1	-	-	14-Week gestation to term	FeSO_4_	60	OD	Placebo
Cogswell *et al*. (2003)	RCT	USA (Ohio)	Low income	24.4	110	86	1 AND ferritin≥20 μg/l	-	1	129 (9)	127 (10)	<20-Week gestation to 28-week gestation	FeSO_4_	30	OD	Placebo
Makrides *et al*. (2003)	RCT	Australia	-	28.3	200	193	1 ANDferritin≥12 μg/l	1 or 2	3	131 (8)	130 (8)	20-Week gestation to term	FeSO_4_	20	OD	Placebo
Siega-Riz *et al*. (2006)	RCT	USA (North Carolina)	-	-	160	156	1 ANDferritin≥40 μg/l	-	1	124 (9)	124 (9)	<20-Week gestation to 26- to 29-week gestation	FeSO_4_	30	OD	Placebo
Ziaei *et al*. (2007)	RCT	Iran	-	25.7	370	357	Hb≥13.2g/dl	-	1	139.8 (5.6)	140.1 (6.2)	13-Week gestation to term	FeSO_4_	50	OD	Placebo
Harvey *et al*. (2007)	RCT	England	-	29.8	6	7	Hb≥10.8 g/dl	Mixed	1	123 (7)	123 (8)	16-Week gestation to term	Ferrous gluconate	100	OD	Placebo
Ziaei *et al*. (2008)	RCT	Iran	-	26.3	100	105	Hb≥13.2g/dl AND ferritin≥15μg/l	Mixed	1	139.9 (5.5)	139.4 (5)	20-Week gestation to term	FeSO_4_	50	OD	Placebo
Ozyigit *et al*. (2008)	Observational	Turkey	-	26.2	35	23	Hb>10g/dl	Primiparous	-	123 (12)	127 (-)	‘At least 2 months’	‘Elemental iron’ (NI)	40	OD	Non-supplemented
Falahi *et al*. (2011)	RCT	Iran	-	23.8	70	78	1 ANDferritin>12 μg/l	Primiparous	-	129.8 (10)	130.5 (8.9)	<20-Week gestation to term	FeSO_4_	60	OD	Placebo
Ouladsahebmadarek *et al*. (2011)	RCT	Iran	-	25.9	410	372	Hb>120g/l	-	1	138.3 (7.8)	132.6 (7.8)	13-Week gestation to term	‘Elemental iron’ (NI)	30	OD	Placebo
Parisi *et al*. (2017)	RCT	Italy	-	30.7	45	12	Hb≥10.5g/dl AND ferritin≥15 μg/l	-	1	119.3 (5.9)^d^	120 (6)	11- to 13-week gestation to 6-week postpartum	FeSO_4_ or ‘liposomal iron’	30, 14, or 28	OD	Non-supplemented
Abioye *et al*. (2023)	RCT	Tanzania	-	-	-	-	1 ANDferritin>12μg/l	1 or 2	3	^c^	^c^	<28-Week gestation to term	FeSO_4_	60	OD	Placebo
Korkmaz *et al*. (2014)	RCT	Turkey	-	24.4	36	36	Hb≥11g/l	Mixed	1	-	-	6-Week gestation to term	(NI)	60	OD	Placebo
Jafarbegloo *et al*. (2015)	RCT	Iran	-	26.2	88	88	Hb>13.2g/dl	Primiparous	1	139 (7.3)	140.1 (7.9)	20-Week gestation to term	FeSO_4_	15^a^	OD	Placebo
Alizadeh *et al*. (2016)	RCT	Iran	-	26.1	32	32	Hb>13.2g/dl AND ferritin>15 μg/l	Mixed	1	136.9 (4.4)	135.7 (4)	16- to 20-week gestation to 12-week postpartum	FeSO_4_	50	OD	Placebo
Tholin *et al*. (1995)	RCT	Denmark	-	25.9	25	19	1 ANDferritin>10μg/l	-	1	^c^	^c^	20-Week gestation to term	FeSO_4_	100	OD	Placebo
Milman (2023)	RCT	Denmark	-	26.6	44	36	1 ANDferritin>30μg/l	Mixed	1	121 (7.1)	123 (6.9)	14- to 18-week gestation to 8-week postpartum	Ferrous fumarate	66	OD	Placebo

- indicates no information provided. NI iron type not specifically indicated. BD, twice daily; OD, once daily; TD, thrice daily.

^a^Elemental iron value not directly stated, therefore calculated (1 mg FeSO_4_ contains ~0.3 mg elemental iron).

^b^Study conducted with two separate groups: A = Antwerp, R = Rotterdam.

^c^Only median value available.

^d^Combined 3 means and SD using Repeating Cochrane’s formula.

Iron was predominantly given as ferrous sulphate, with varying doses (eight studies used ≤30 mg, eight used between 50 and 66 mg, and six studies used ≥100 mg). Some older studies reported extremely high elemental iron doses that may instead represent compound doses [26, 27, 29, 47]. Their data were reported as published, but separated during subgroup analysis of dose. One study [43] initiated supplementation later than 20 weeks gestation. Two studies [33, 35] stopped supplementation before term.

### Risk of bias assessment

**Figure 2.**
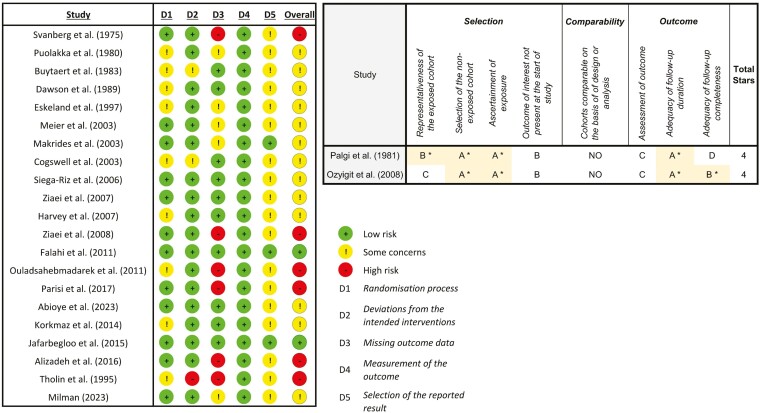
(a) ROB2 assessment (left) and (b) Newcastle-Ottawa Scale (right).

A risk of bias assessment was conducted for the 21 RCTs (Fig. 2). Overall risk was deemed ‘Low’ for 2 studies, ‘Some concerns’ for 13 studies, and ‘High’ for 6 studies. Many studies intervened if participant Hb dropped too low. Several studies excluded these data, introducing a high degree of bias in favour of the null hypothesis [26, 41, 42, 46, 47]. Other studies analysed these participants in their original groups, reducing the apparent effect of the intervention [32, 33]. One study excluded participants based on birth outcomes [38] introducing selection bias. The most common reason for ‘some concerns’ was the absence of study preregistration.

Both observational studies [28, 39] were deemed ‘fair quality’ using the Newcastle-Ottawa Scale (Fig. 2b).

### Benefits of supplementation

#### Haemoglobin

**Figure 3.**
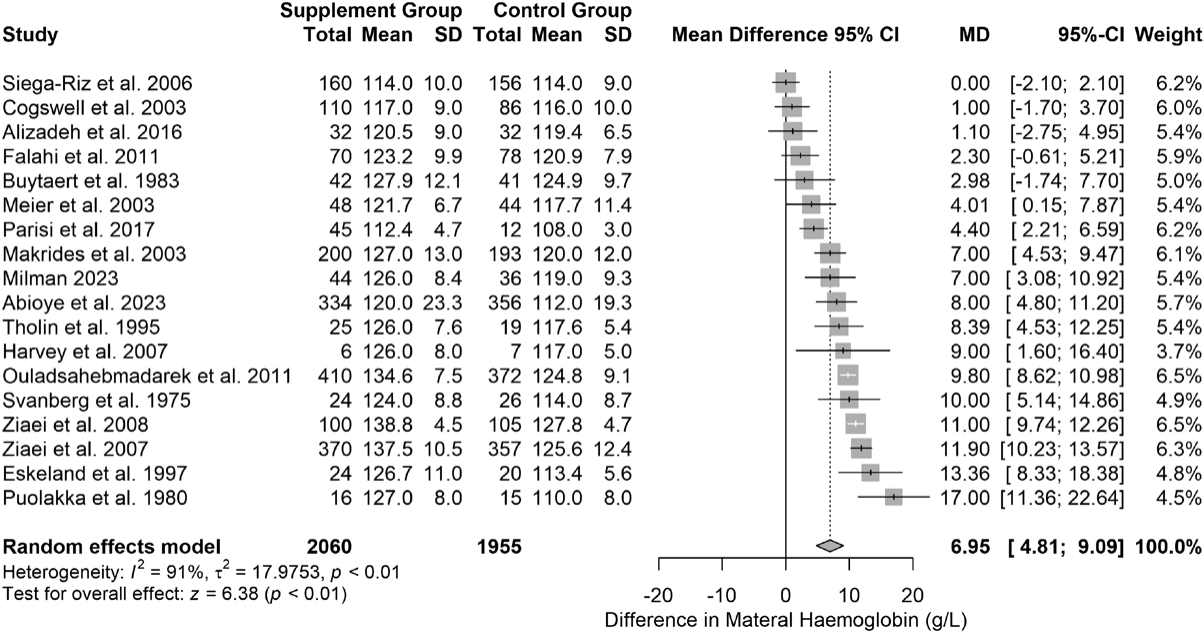
Effect of iron supplementation on maternal haemoglobin. Forest plot showing the MDs in maternal haemoglobin between iron- and non-supplemented groups across 18 studies (*n* = 4015). Haemoglobin was higher in the supplemented group (MD = 6.95 g/l, 95% CI = 4.81–9.09, *P* < .01). There was a high degree of heterogeneity (*I*^2^ = 91%, tau^2^ = 17.97) but the direction of effect was consistent.

Eighteen studies reported maternal haemoglobin (*n* = 4015). Haemoglobin was higher in the supplemented group (MD = 6.95 g/l, 95% CI: 4.81–9.09, *P* < .001) (Fig. 3). There was high heterogeneity (*I*^2^ = 91%, tau^2^ = 17.97) but the direction of effect was consistent. The certainty of evidence was moderate (downgraded one level due to inconsistency, owing to high heterogeneity but consistent direction of effect). Sensitivity analysis suggested the impact of bias was small (MD = 6.69 g/l, 95% CI: 3.78–9.61, *P* < .001) (Supplementary Fig. S2), thus the certainty of evidence rating was not downgraded due to risk of bias. Subgroup analysis based on dose, the authors’ anaemia definition, supplement start time and HDI were not informative (Supplementary Figs S3–S6). Studies measuring Hb before 34 weeks found smaller effects than studies measuring at term or after 34 weeks (Chi^2^ = 14.55, *P* < .01) (Supplementary Fig. S7). Based on the prediction interval for this analysis, we cannot exclude the possibility of future studies finding an MD between −2.33 and 16.23 g/l.

#### Maternal anaemia

**Figure 4.**
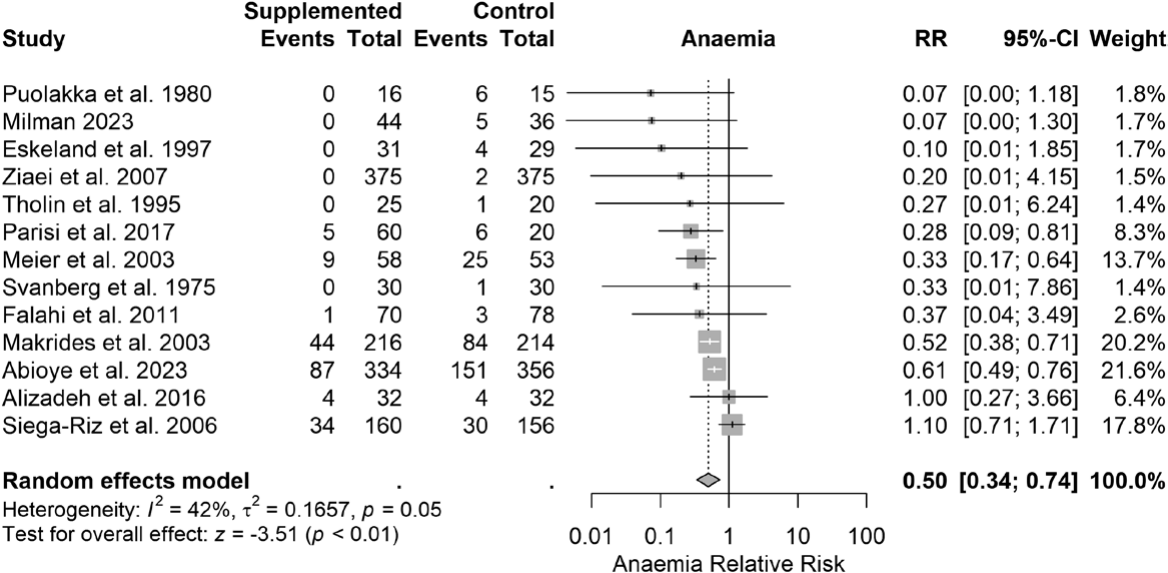
Effect of iron supplementation on risk of maternal anaemia. Forest plot showing the RR of maternal anaemia between iron- and non-supplemented groups across 13 studies (*n* = 2315). The risk of anaemia was lower in the supplemented group (RR = 0.50, 95% CI = 0.34–0.74, *P* < .01). There was a moderate degree of heterogeneity (*I*^2^ = 42%, tau^2^ = 0.17).

Thirteen studies reported the rate of maternal anaemia (*n* = 2315). In the supplemented groups 128/1185 participants (10.8%) developed anaemia, compared with 231/1130 participants (20.4%) in the control groups (RR = 0.50, 95% CI: 0.34–0.74, *P* < .001) (Fig. 4). Therefore for every 1000 women given iron supplementation, 102 fewer cases of anaemia occurred. Heterogeneity was moderate (*I*^2^ = 42%, tau^2^ = 0.17). The certainty of evidence was high. Sensitivity analysis suggested the impact of bias was small (RR = 0.50, 95% CI: 0.32–0.79, *P* < .001) (Supplementary Fig. S8), thus the certainty of evidence rating was not downgraded due to the risk of bias. The impact of zero count cell corrections was also small (odds ratio = 0.33, 95% CI: 0.20–0.56, *P* < .001) (Supplementary Fig. S9). Subgroup analyses were not informative (Supplementary Figs S10–S13). On visual inspection, the studies with high standard error were asymmetrically distributed, suggesting possible publication bias (Supplementary Fig. S14). However, excluding smaller studies had little effect on the effect estimate (RR = 0.58, 95% CI: 0.39–0.88, *P* < .03), thus the certainty of evidence rating was not downgraded due to publication bias (Supplementary Fig. S15). Based on the prediction interval for this analysis, we cannot exclude the possibility of future studies finding an RR between 0.19 and 1.36.

#### Maternal ferritin

Fourteen studies reported maternal ferritin (*n*=2853). Ferritin was higher in the supplemented groups (MD = 12.22 ng/ml, 95% CI: 6.92–17.52, *P* < .001) (Supplementary Fig. S16). There was a high degree of heterogeneity (*I*^2^ = 87%, tau2 = 87.95) but the direction of effect was consistent. The certainty of evidence was moderate (downgraded one level due to inconsistency, owing to high heterogeneity but consistent direction of effect). Sensitivity analysis suggested the impact of bias was small (MD = 14.98 ng/ml, 95% CI: 7.23–22.73, *P* < .001) (Supplementary Fig. S17), thus the certainty of evidence rating was not downgraded due to risk of bias. Subgroup analysis based on dose, the authors’ anaemia definition, and supplement start time were not informative (Supplementary Figs S18–S20). Studies measuring ferritin before 34 weeks had smaller effects than studies measuring at term or after 34 weeks (Chi^2^ = 9.14, *P* = .01) (Supplementary Fig. S21). Studies conducted in nations with high and very high HDI found lower increases in ferritin than the study conducted in a low HDI nation (Chi^2^ = 11.92, *P* < .01) (Supplementary Fig. S22).

#### Birth weight

Thirteen studies reported birth weight (*n* = 2994). There was no statistically significant effect of the intervention (MD = 17.75 g, 95% CI: −55.74 to 91.24, *P* = .64) (Supplementary Fig. S23). There was a high degree of heterogeneity (*I*^2^ = 61%, tau^2^ = 10 221.02) and studies found both positive and negative effects of intervention. The certainty of the evidence was very low (downgraded two levels due to inconsistency, given the inconsistent direction of effect; downgraded one level due to risk of bias, as several high risk of bias studies were included in the analysis). Subgroup analysis was uninformative (Supplementary Figs S24–S27).

#### Caesarean sections

Five studies reported non-elective caesarean section rates (*n* = 1552). There was no statistically significant effect of intervention (RR = 1.07, 95% CI: 0.88–1.29, *P* = .51) (Supplementary Fig. S28). The certainty of the evidence was low (downgraded one level due to imprecision, as there were fewer than 2000 participants; downgraded one level due to high risk of bias, as several high-risk studies were included in the analysis).

#### Preterm births

Five studies reported on rates of preterm birth (*n* = 2204). There was no statistically significant effect of intervention (RR = 0.82, 95% CI: 0.58–1.17, *P* = .28) (Supplementary Fig. S29). The certainty of evidence was moderate (downgraded one level due to high risk of bias, as several high-risk studies were included in the analysis).

### Harms of supplementation

#### Gastrointestinal side effects

Four studies reported gastrointestinal (GI) side effects [28, 32, 34, 45] (Supplementary Table S30), including nausea, vomiting, constipation, diarrhoea, abdominal pain, loss of appetite, and heartburn. Palgi *et al.* [28] was the only observational study reporting harms but did not report the rate of side effects in the control group. The other studies did not report a statistically significant effect of intervention for any GI side effect.

#### Other side effects

Seventeen studies reported at least one side effect (15 RCTs and 2 observational studies—for a full list, see Supplementary Table S30). Three studies reported on zinc metabolism [30, 34, 37], two on size of infant for gestational age [33, 36] (both finding significant effects in opposing directions), one on gestational diabetes [48], and six on average gestational age at delivery [30, 32, 34, 35, 40, 42]. One study found an association between iron- and pregnancy-induced hypertension [41] and another between iron and oligohydramnios [44].

## Discussion

### Summary of findings

Prophylactic iron supplementation likely results in a large reduction in maternal anaemia during pregnancy. Despite a high degree of statistical heterogeneity, most studies found that supplementation improved haematological indices at term. Patient-important outcomes, such as quality of life, fatigue and depression, were not reported. Foetal outcomes were better represented, with birth weight, preterm birth, and average gestational age being explored in multiple studies. However, we did not identify an association between supplementation and improved foetal outcomes. Few included studies explored maternal harms and there was insufficient evidence to comment further on an association between iron supplements and GI disturbance or iron overload-related complications.

### Findings in the context of current literature

A 2015 systematic review found that iron supplementation greatly increased maternal haemoglobin [14]. However, the review did not differentiate participants based on initial haemoglobin status and it is unclear whether this benefit was confined only to anaemic women. Given the well-established benefit of iron supplementation for anaemic women, it is unsurprising that the average effect of supplementation across the whole pregnant population was positive. We have shown that the benefits of iron supplementation also extend to non-anaemic women. This benefit may be explained by a subpopulation of women who are iron deficient at the start of pregnancy but are not yet anaemic [49]. For these women, iron supplementation could prevent progression to anaemia by treating the underlying deficiency. If this is the case, these women represent a clinically important population who are currently undetected and untreated in countries where antenatal iron status is not routinely assessed. Many women do not receive sufficient dietary iron to meet the increased requirements of pregnancy [50] and ID can arise even among initially iron-replete women. A 2023 systematic review found iron supplements reduce anaemia even for women who are not iron deficient at the start of their pregnancy [15]. Therefore, antenatal screening of iron status may be insufficient to identify all women who could benefit from iron supplements. One solution is to stratify patients by risk factors for ID, such as multiple pregnancies, diet, and age when recommending iron supplements [51]. However, there is no evidence to suggest this strategy maximizes the benefits of iron supplementation.

A 2015 systematic review found a clear relationship between iron supplementation and GI-specific side effects in non-pregnant populations [18]. However, these findings may not translate to pregnant populations due to the increased iron requirement and altered physiology of pregnancy. An RCT studying non-anaemic pregnant women found the prevalence of GI disturbance did not differ when taking iron supplements. The authors suggested that any perceived side effects of iron supplements may instead be due to physiological changes in pregnancy [45]. Our findings, based on the current evidence base, are inadequate to conclude whether or not an association exists between iron supplementation and GI disturbance in pregnancy.

Other proposed side effects of iron supplementation, such as preeclampsia, prematurity, and foetal growth restriction, could result from iron overload [16, 17]. Conditions such as hereditary haemochromatosis may increase the risk of iron overload due to excessive iron absorption [10]. For people without haemochromatosis, it is unclear how iron supplementation could cause iron overload in the context of normal homeostatic regulation of enteral iron absorption. The studies included in our review were inconsistent in their reporting of side effects, thus there is limited evidence to conclude on this topic.

### Limitations of the review

This review was inspired by the author’s encounters with patients in primary care, which highlighted the importance of the topic. However, patients were not specifically involved in the design and interpretation of the study. Therefore, it is possible that outcomes important to patients were missed. For example, we focussed on once-daily iron intake as this is the most common dosing regimen, however, this may not align with real-world preferences. This limitation was partially mitigated by including all reported outcomes from included studies, however, future research should be co-designed with patients.

Researchers had an ethical obligation to treat participants in the control group if they became anaemic during the trial. Some studies excluded these participants, which artificially increased the control groups’ haemoglobin. Other studies analysed these participants in their original group, which may have obscured differences between control and intervention groups. Consequently, our analysis may underestimate both the benefits and harms of iron supplementation, though this effect is probably less important given the high variability observed between studies.

To maximize the quantity of data included in this review, a wide range of studies were included. This introduced methodological heterogeneity, with substantial differences in variables such as dose, duration, and authors’ definitions of anaemia. These differences may have altered the true effect of intervention in each study, so the combined effect estimate may not correspond to a clinically relevant intervention regime. Indeed, the duration of supplementation seems to influence the benefit, with greater differences in haemoglobin seen later in pregnancy. Therefore, the combined effect estimate for this outcome should not be considered an expectation for haemoglobin rise in any specific population. In contrast, supplement dose is likely to be a less significant source of methodological heterogeneity since low-dose iron has been shown to provide the same reduction in anaemia as higher doses [52]. Given the direction of effect in most outcomes was consistent, the overall effect of methodological heterogeneity on the effect estimate was likely to be small. There was, however, substantial variation in how studies reported on harms, highlighting a need for standardized outcome measures to strengthen the consistency and quality of evidence.

Non-anaemic pregnant women are heterogeneous with respect to iron status, which may impact the measured intervention effect. Included studies were conducted in different countries, which may have differences in baseline ID risk. Therefore, our combined effect estimate may average across distinct populations, who experienced disparate levels of benefit and harm. HDI was used as a rough proxy for risk of ID but it does not account for all the potentially relevant clinical variables. Included studies did not report sufficient participant information to interrogate the different sources of clinical heterogeneity in more detail. Therefore, we were unable to identify any specific clinical factors that might influence the magnitude of benefit and harm in non-anaemic populations.

### Implications for future research, policy, and practice

While there is clear evidence that iron supplements benefit non-anaemic pregnant women, haematological benefits are only clinically relevant if they provide tangible improvements in quality of life. None of the included studies reported on patient-important outcomes such as fatigue and depression despite evidence that iron supplements improve these outcomes in the anaemic population [53]. Future research should focus on quantifying changes in both physical and mental health-related quality of life.

In order to determine which women experience the most benefit from iron supplementation, future studies should create comparison groups based on initial iron status or risk factors for ID such as dietary iron consumption. Studies should collect and report a wide range of participant information to allow for exploratory subgroup analysis and facilitate individual participant data meta-analysis. By clarifying the sources of clinical heterogeneity, it may be possible to create and validate models to predict benefit. This would allow guidelines to integrate evidenced-based risk stratification and maximize the benefit of supplements. Similarly, researchers should explore which women experience the most harm from iron supplements. There is a general paucity of evidence surrounding the harms of iron supplementation which needs to be addressed to inform guidelines. Research should focus on GI disturbances and the potential for iron overload as these harms are commonly mentioned in the literature and may have a large impact on maternal quality of life.

Finally, the variety of supplement regimens in our review reflects an uncertainty in the literature regarding the optimal dosage of iron supplementation. For the anaemic population, there is ongoing research aiming to identify the regimen with maximal benefit and minimal harm [54]. However, similar research for non-anaemic populations has not yet been conducted.

## Conclusion

Iron supplements likely benefit non-anaemic pregnant women. The size of this benefit will vary based on individual context, and the impact on quality of life remains to be quantified. However, we estimate for every ten women taking iron, one fewer will develop anaemia. In contrast, there is insufficient evidence to quantify the harms of iron supplements in the same population. Therefore, this review highlights four important avenues for future research; quantification of harms, optimization of dosing regimens, determining the clinical relevance of any benefit, and identifying subpopulations with disparate outcomes. Exploring these areas will enable women, clinicians and guideline developers to make informed decisions regarding prophylactic iron supplementation.

## Supplementary Material

cmae079_suppl_Supplementary_Figures_S1-S29_Tables_S30

## Data Availability

The data supporting the findings of this systematic review are openly available in the published reports of the included studies. All data generated or analysed during this study are included in this published article (and its supplementary information files).
