# Supplementary material for: The benefits and harms of oral iron supplementation in non-anaemic pregnant women: a systematic review and meta-analysis
Source: Fam Pract. 2025 Jan 21;42(1):cmae079. doi: 10.1093/fampra/cmae079 (PMC11747145; doi:10.1093/fampra/cmae079)
Supplement: cmae079_suppl_Supplementary_Figures_S1-S29_Tables_S30 [file cmae079_suppl_supplementary_figures_s1-s29_tables_s30.pdf]

Supplementary Materials

Figure S1 - Table of search strategies. MeSH terms used for Medline and Cochrane library searches. Emtree used for Embase searches.

	Medline (via Ovid)	Embase (via Ovid)	Cochrane Library
1	pregnancy/ or labour onset/ or term birth/ or exp pregnancy outcome/ or prenatal nutritional physiological phenomena/ or pregnant women/	pregnancy/ or pregnancy outcome.mp.	[mh ^pregnancy] OR [mh ^"labor onset"] OR [mh ^"term birth"] OR [mh "pregnancy outcome"] OR [mh ^"prenatal nutritional physiological phenomena"] OR [mh ^"pregnant women"]
2	Child?bearing or child?carrying or pregnan* or maternal	child bearing.mp.	(child?bearing:ti OR child?carrying:ti OR pregnan*:ti OR maternal:ti)
3	1 or 2	1 or 2	#1 OR #2
4	Iron Deficiencies/	Iron deficiency/ or iron deficiency.mp.	[mh ^"Iron Deficiencies"]
5	iron deficien* or ID or IDWA or NAID or iron replete	Non anaemic.mp.	(("iron" NEXT deficien*):ti,ab,kw OR ID:ti,ab,kw OR IDWA:ti,ab,kw OR NAID:ti,ab,kw OR "iron replete":ti,ab,kw)
6	Nonan?emic or non-an?emic	ID.mp. or IDWA.mp. or NAID.mp. or iron replete.mp.	(nonan?emic:ti,ab,kw OR non-an?emic:ti,ab,kw)
7	4 or 5 or 6	4 or 5 or 6	#4 OR #5 OR #6
8	Iron/ad	iron/ or iron.mp.	[mh ^Iron]
9	Iron supplement* or iron adj3 multivitamin or iron adj3 vitamin	Iron supplement.mp. Or iron therapy/	(("iron" NEXT supplement*):ti,ab,kw OR (iron:ti,ab,kw NEAR/3 multivitamin:ti,ab,kw) OR (iron:ti,ab,kw NEAR/3 vitamin:ti,ab,kw))
10	8 or 9	8 or 9	#8 OR #9
11	3 and 7 and 10	3 and 7 and 10	(#3 AND #7 AND #10) NOT ([mh animals] NOT [mh ^humans])
12	11 not (exp animals/ not humans)	11 not ((exp animal/ or exp invertebrate/ or nonhuman/ or animal experiment/ or animal tissue/ or animal model/ or exp plant/ or exp fungus/) not (exp human/ or human tissue/))	

	clinicaltrials.gov	ICTRP (https://trialsearch.who.int/)
Title		(Iron Deficiency or Iron Deficiency Anaemia) and pregnancy
Intervention	Iron or Iron supplement	Iron or Iron supplement
Condition	Iron Deficiency or Iron Deficiency Anaemia	Iron Deficiency or Iron Deficiency Anaemia
Other terms	pregnancy	
Study status	Any	Any
Sex	Female	
Age	Adult (18-64)	Adult (18-64)
Date of registration	Any	Any
Study type	Observational or Randomised	Observational or Randomised

Figure S2 - Forest plot showing the effect of iron supplementation on maternal haemoglobin excluding studies with high risk of bias.

Study	Supplement Group Total	Supplement Group Mean	Supplement Group SD	Control Group Total	Control Group Mean	Control Group SD	MD	95%-CI	Weight
Siega-Riz et al. 2006	160	114.0	10.0	156	114.0	9.0	0.00	[-2.10; 2.10]	9.3%
Cogswell et al. 2003	110	117.0	9.0	86	116.0	10.0	1.00	[-1.70; 3.70]	9.0%
Falahi et al. 2011	70	123.2	9.9	78	120.9	7.9	2.30	[-0.61; 5.21]	8.9%
Buytaert et al. 1983	42	127.9	12.1	41	124.9	9.7	2.98	[-1.74; 7.70]	7.8%
Meier et al. 2003	48	121.7	6.7	44	117.7	11.4	4.01	[0.15; 7.87]	8.4%
Makrides et al. 2003	200	127.0	13.0	193	120.0	12.0	7.00	[4.53; 9.47]	9.2%
Milman 2023	44	126.0	8.4	36	119.0	9.3	7.00	[3.08; 10.92]	8.3%
Abioye et al. 2023	334	120.0	23.3	356	112.0	19.3	8.00	[4.80; 11.20]	8.8%
Harvey et al. 2007	6	126.0	8.0	7	117.0	5.0	9.00	[1.60; 16.40]	6.0%
Ziaei et al. 2007	370	137.5	10.5	357	125.6	12.4	11.90	[10.23; 13.57]	9.5%
Eskeland et al. 1997	24	126.7	11.0	20	113.4	5.6	13.36	[8.33; 18.38]	7.6%
Puolakka et al. 1980	16	127.0	8.0	15	110.0	8.0	17.00	[11.36; 22.64]	7.2%
**Random effects model**	**1424**			**1389**			**6.69**	**[3.78; 9.61]**	**100.0%**
**Prediction interval**								**[-4.39; 17.78]**	

Heterogeneity: $I^2 = 91\%$, $\tau^2 = 22.5559$, $p < 0.01$

Test for overall effect: $z = 4.50$ ($p < 0.01$)

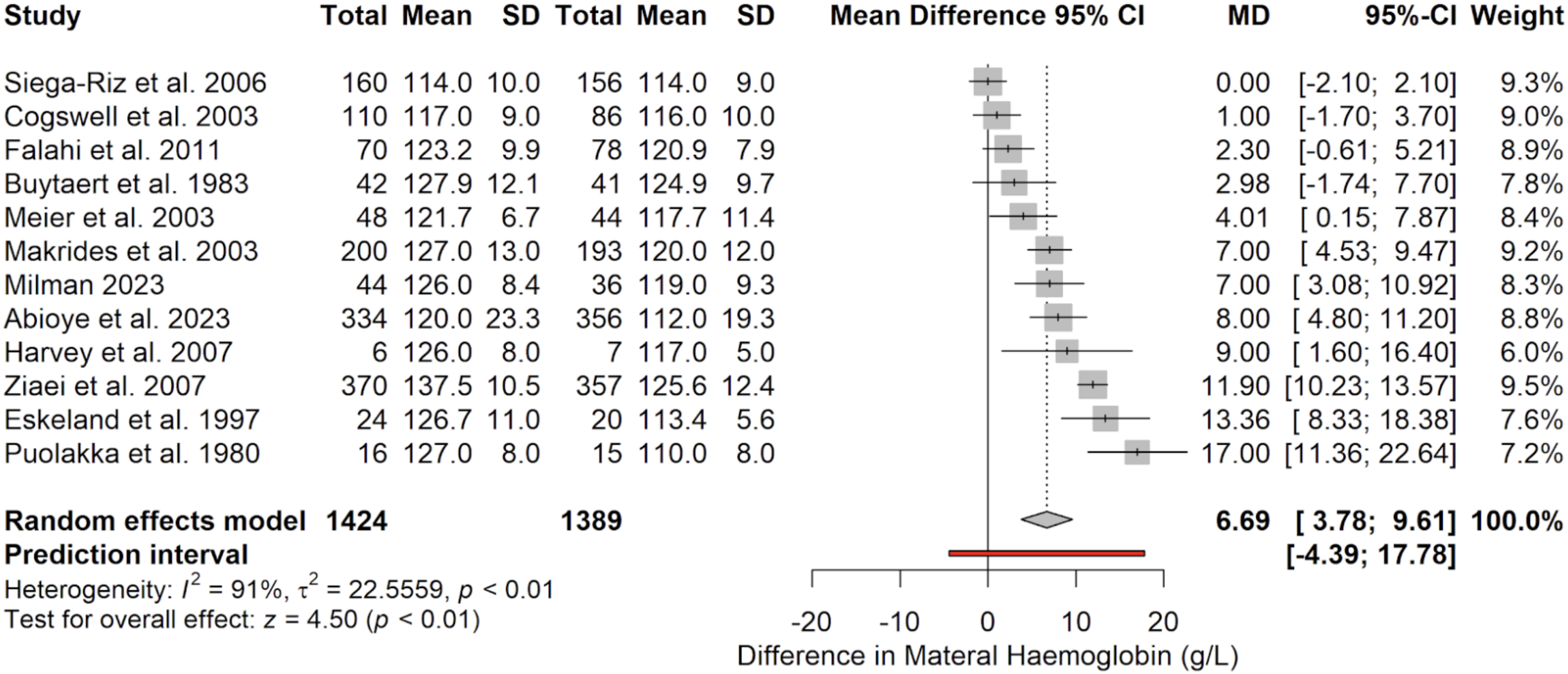

Figure S3 - Forest plot showing the effect of iron supplementation on maternal haemoglobin with subgroup analysis based on supplement dose.

Study	Supplement Group Total	Supplement Group Mean	Supplement Group SD	Control Group Total	Control Group Mean	Control Group SD	MD	95%-CI
**Dose = Extreme**								
Buytaert et al. 1983	42	127.9	12.1	41	124.9	9.7	2.98	[-1.74; 7.70]
Tholin et al. 1995	25	126.0	7.6	19	117.6	5.4	8.39	[4.53; 12.25]
Harvey et al. 2007	6	126.0	8.0	7	117.0	5.0	9.00	[1.60; 16.40]
Svanberg et al. 1975	24	124.0	8.8	26	114.0	8.7	10.00	[5.14; 14.86]
Puolakka et al. 1980	16	127.0	8.0	15	110.0	8.0	17.00	[11.36; 22.64]
**Random effects model**	**113**			**108**			**9.33**	**[4.91; 13.75]**
**Prediction interval**								**[-6.03; 24.69]**
Heterogeneity: $I^2 = 72\%$, $\tau^2 = 18.1982$, $p < 0.01$								
**Dose = Low**								
Siega-Riz et al. 2006	160	114.0	10.0	156	114.0	9.0	0.00	[-2.10; 2.10]
Cogswell et al. 2003	110	117.0	9.0	86	116.0	10.0	1.00	[-1.70; 3.70]
Parisi et al. 2017	45	112.4	4.7	12	108.0	3.0	4.40	[2.21; 6.59]
Makrides et al. 2003	200	127.0	13.0	193	120.0	12.0	7.00	[4.53; 9.47]
Ouladsahebmadarek et al. 2011	410	134.6	7.5	372	124.8	9.1	9.80	[8.62; 10.98]
Eskeland et al. 1997	24	126.7	11.0	20	113.4	5.6	13.36	[8.33; 18.38]
**Random effects model**	**949**			**839**			**5.73**	**[1.79; 9.68]**
**Prediction interval**								**[-8.53; 20.00]**
Heterogeneity: $I^2 = 95\%$, $\tau^2 = 22.3411$, $p < 0.01$								
**Dose = High**								
Alizadeh et al. 2016	32	120.5	9.0	32	119.4	6.5	1.10	[-2.75; 4.95]
Falahi et al. 2011	70	123.2	9.9	78	120.9	7.9	2.30	[-0.61; 5.21]
Meier et al. 2003	48	121.7	6.7	44	117.7	11.4	4.01	[0.15; 7.87]
Milman 2023	44	126.0	8.4	36	119.0	9.3	7.00	[3.08; 10.92]
Abioye et al. 2023	334	120.0	23.3	356	112.0	19.3	8.00	[4.80; 11.20]
Ziaei et al. 2008	100	138.8	4.5	105	127.8	4.7	11.00	[9.74; 12.26]
Ziaei et al. 2007	370	137.5	10.5	357	125.6	12.4	11.90	[10.23; 13.57]
**Random effects model**	**998**			**1008**			**6.69**	**[3.51; 9.88]**
**Prediction interval**								**[-4.43; 17.82]**
Heterogeneity: $I^2 = 91\%$, $\tau^2 = 16.0750$, $p < 0.01$								
Heterogeneity: $I^2 = 91\%$, $\tau^2 = 17.9753$, $p < 0.01$								
Test for subgroup differences: $\chi^2_2 = 1.50$, df = 2 ($p = 0.47$)								

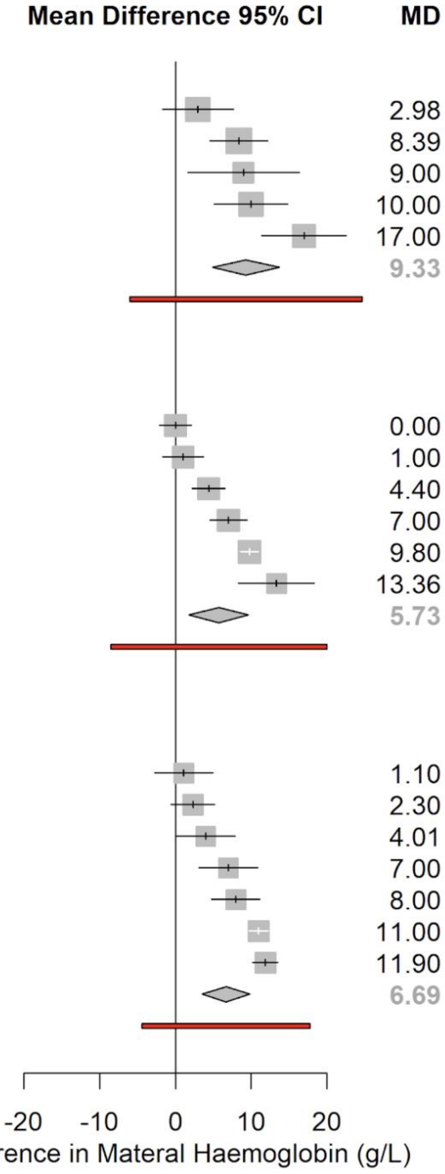

Figure S4 - Forest plot showing the effect of iron supplementation on maternal haemoglobin with subgroup analysis based on the study's definition of anaemia in their inclusion criteria. As compared to the WHO definition of less than 110g/L.

Study	Supplement Group Total	Supplement Group Mean	Supplement Group SD	Control Group Total	Control Group Mean	Control Group SD	MD	95%-CI
**Definition_Hb = Higher**								
Alizadeh et al. 2016	32	120.5	9.0	32	119.4	6.5	1.10	[-2.75; 4.95]
Buytaert et al. 1983	42	127.9	12.1	41	124.9	9.7	2.98	[-1.74; 7.70]
Ouladsahebmadarek et al. 2011	410	134.6	7.5	372	124.8	9.1	9.80	[8.62; 10.98]
Svanberg et al. 1975	24	124.0	8.8	26	114.0	8.7	10.00	[5.14; 14.86]
Ziaei et al. 2008	100	138.8	4.5	105	127.8	4.7	11.00	[9.74; 12.26]
Ziaei et al. 2007	370	137.5	10.5	357	125.6	12.4	11.90	[10.23; 13.57]
**Random effects model**	**978**			**933**			**8.10**	**[4.57; 11.63]**
**Prediction interval**								**[-4.35; 20.56]**
Heterogeneity: $I^2 = 86\%$, $\tau^2 = 16.8799$, $p < 0.01$								
**Definition_Hb = 110**								
Siega-Riz et al. 2006	160	114.0	10.0	156	114.0	9.0	0.00	[-2.10; 2.10]
Cogswell et al. 2003	110	117.0	9.0	86	116.0	10.0	1.00	[-1.70; 3.70]
Falahi et al. 2011	70	123.2	9.9	78	120.9	7.9	2.30	[-0.61; 5.21]
Meier et al. 2003	48	121.7	6.7	44	117.7	11.4	4.01	[0.15; 7.87]
Makrides et al. 2003	200	127.0	13.0	193	120.0	12.0	7.00	[4.53; 9.47]
Milman 2023	44	126.0	8.4	36	119.0	9.3	7.00	[3.08; 10.92]
Abioye et al. 2023	334	120.0	23.3	356	112.0	19.3	8.00	[4.80; 11.20]
Tholin et al. 1995	25	126.0	7.6	19	117.6	5.4	8.39	[4.53; 12.25]
Eskeland et al. 1997	24	126.7	11.0	20	113.4	5.6	13.36	[8.33; 18.38]
Puolakka et al. 1980	16	127.0	8.0	15	110.0	8.0	17.00	[11.36; 22.64]
**Random effects model**	**1031**			**1003**			**6.46**	**[3.31; 9.61]**
**Prediction interval**								**[-5.07; 17.98]**
Heterogeneity: $I^2 = 87\%$, $\tau^2 = 22.3877$, $p < 0.01$								
**Definition_Hb = Lower**								
Parisi et al. 2017	45	112.4	4.7	12	108.0	3.0	4.40	[2.21; 6.59]
Harvey et al. 2007	6	126.0	8.0	7	117.0	5.0	9.00	[1.60; 16.40]
**Random effects model**	**51**			**19**			**5.29**	**[1.73; 8.85]**
**Prediction interval**								
Heterogeneity: $I^2 = 27\%$, $\tau^2 = 2.8374$, $p = 0.24$								
Heterogeneity: $I^2 = 91\%$, $\tau^2 = 17.9753$, $p < 0.01$								
Test for subgroup differences: $\chi^2_2 = 1.23$, df = 2 ($p = 0.54$)								

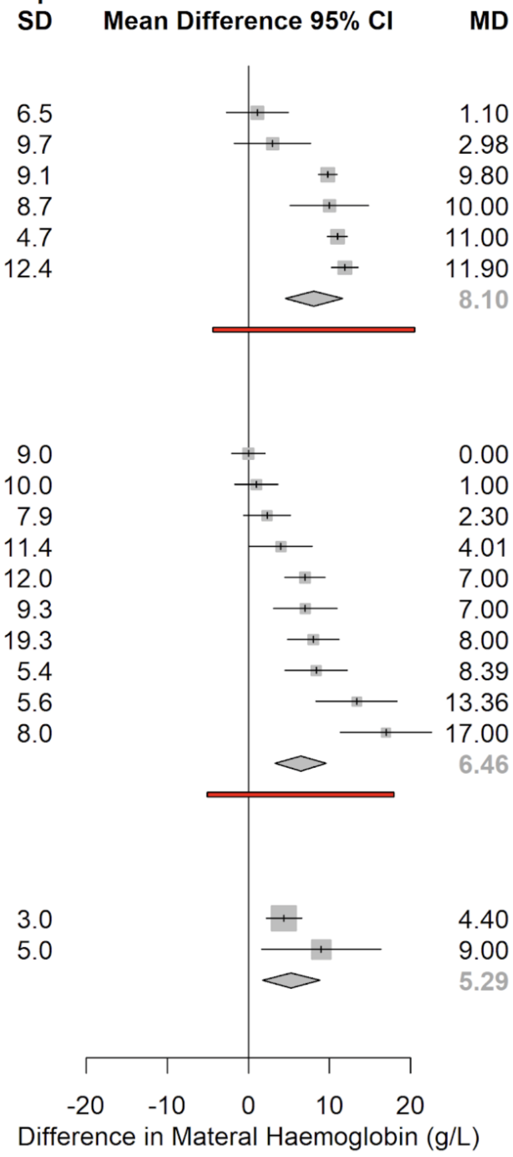

Figure S5 - Forest plot showing the effect of iron supplementation on maternal haemoglobin with subgroup analysis based on when during pregnancy iron supplements were started.

Study	Supplement Group Total	Supplement Group Mean	Supplement Group SD	Control Group Total	Control Group Mean	Control Group SD	MD	95%-CI
**Start = Second Trimester**								
Siega-Riz et al. 2006	160	114.0	10.0	156	114.0	9.0	0.00	[-2.10; 2.10]
Cogswell et al. 2003	110	117.0	9.0	86	116.0	10.0	1.00	[-1.70; 3.70]
Alizadeh et al. 2016	32	120.5	9.0	32	119.4	6.5	1.10	[-2.75; 4.95]
Falahi et al. 2011	70	123.2	9.9	78	120.9	7.9	2.30	[-0.61; 5.21]
Buytaert et al. 1983	42	127.9	12.1	41	124.9	9.7	2.98	[-1.74; 7.70]
Meier et al. 2003	48	121.7	6.7	44	117.7	11.4	4.01	[0.15; 7.87]
Makrides et al. 2003	200	127.0	13.0	193	120.0	12.0	7.00	[4.53; 9.47]
Milman 2023	44	126.0	8.4	36	119.0	9.3	7.00	[3.08; 10.92]
Abioye et al. 2023	334	120.0	23.3	356	112.0	19.3	8.00	[4.80; 11.20]
Tholin et al. 1995	25	126.0	7.6	19	117.6	5.4	8.39	[4.53; 12.25]
Harvey et al. 2007	6	126.0	8.0	7	117.0	5.0	9.00	[1.60; 16.40]
Ziaei et al. 2008	100	138.8	4.5	105	127.8	4.7	11.00	[9.74; 12.26]
Puolakka et al. 1980	16	127.0	8.0	15	110.0	8.0	17.00	[11.36; 22.64]
**Random effects model**	**1187**			**1168**			**5.83**	**[3.30; 8.35]**
**Prediction interval**								**[-3.92; 15.57]**
Heterogeneity: $I^2 = 91\%$, $\tau^2 = 17.9267$, $p < 0.01$								
**Start = First Trimester**								
Parisi et al. 2017	45	112.4	4.7	12	108.0	3.0	4.40	[2.21; 6.59]
Ouladsahebmadarek et al. 2011	410	134.6	7.5	372	124.8	9.1	9.80	[8.62; 10.98]
Svanberg et al. 1975	24	124.0	8.8	26	114.0	8.7	10.00	[5.14; 14.86]
Ziaei et al. 2007	370	137.5	10.5	357	125.6	12.4	11.90	[10.23; 13.57]
Eskeland et al. 1997	24	126.7	11.0	20	113.4	5.6	13.36	[8.33; 18.38]
**Random effects model**	**873**			**787**			**9.65**	**[6.58; 12.73]**
**Prediction interval**								**[-1.50; 20.80]**
Heterogeneity: $I^2 = 87\%$, $\tau^2 = 9.8188$, $p < 0.01$								

Heterogeneity: $I^2 = 91\%$, $\tau^2 = 17.9753$, $p < 0.01$

Test for subgroup differences: $\chi^2_1 = 3.56$, df = 1 ($p = 0.06$)

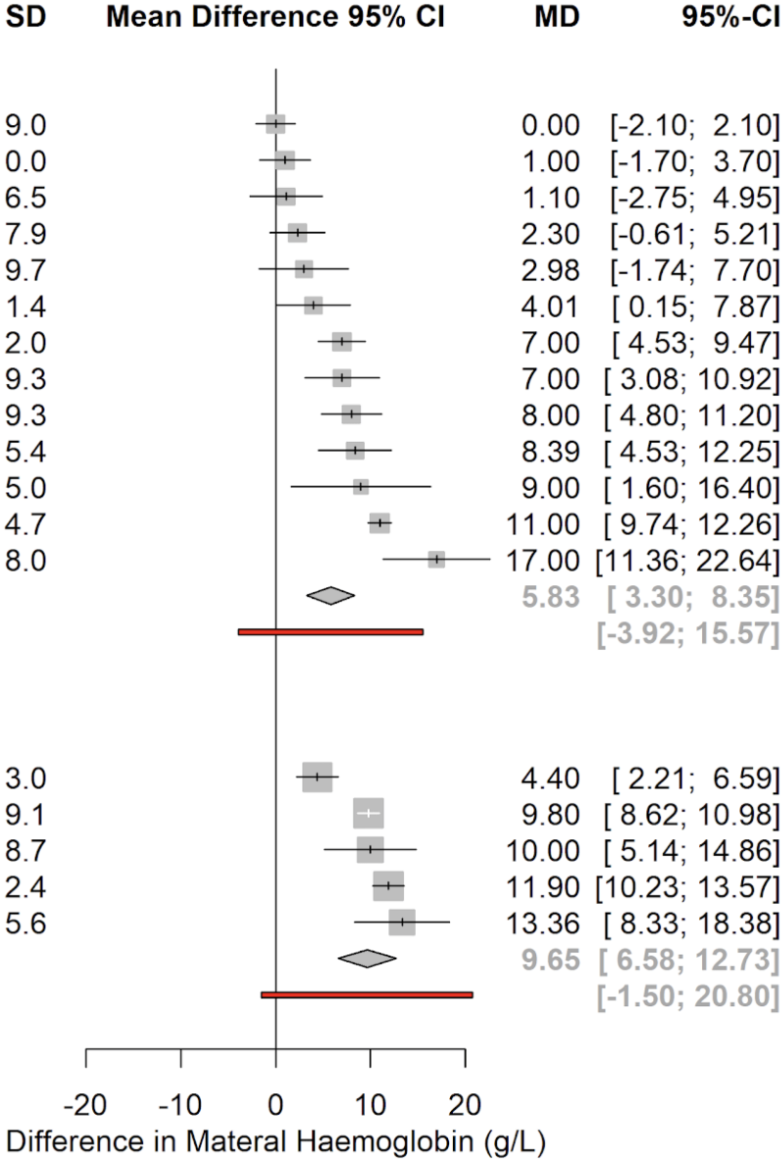

Figure S6 - Forest plot showing the effect of iron supplementation on maternal haemoglobin with subgroup analysis based on human development index in the country and year of the study.

Study	Supplement Group Total	Supplement Group Mean	Supplement Group SD	Control Group Total	Control Group Mean	Control Group SD	MD	95%-CI
**HDI_class = Very High**								
Siega-Riz et al. 2006	160	114.0	10.0	156	114.0	9.0	0.00	[-2.10; 2.10]
Cogswell et al. 2003	110	117.0	9.0	86	116.0	10.0	1.00	[-1.70; 3.70]
Buytaert et al. 1983	42	127.9	12.1	41	124.9	9.7	2.98	[-1.74; 7.70]
Meier et al. 2003	48	121.7	6.7	44	117.7	11.4	4.01	[0.15; 7.87]
Parisi et al. 2017	45	112.4	4.7	12	108.0	3.0	4.40	[2.21; 6.59]
Makrides et al. 2003	200	127.0	13.0	193	120.0	12.0	7.00	[4.53; 9.47]
Milman 2023	44	126.0	8.4	36	119.0	9.3	7.00	[3.08; 10.92]
Tholin et al. 1995	25	126.0	7.6	19	117.6	5.4	8.39	[4.53; 12.25]
Harvey et al. 2007	6	126.0	8.0	7	117.0	5.0	9.00	[1.60; 16.40]
Svanberg et al. 1975	24	124.0	8.8	26	114.0	8.7	10.00	[5.14; 14.86]
Eskeland et al. 1997	24	126.7	11.0	20	113.4	5.6	13.36	[8.33; 18.38]
Puolakka et al. 1980	16	127.0	8.0	15	110.0	8.0	17.00	[11.36; 22.64]
**Random effects model**	**744**			**655**			**6.61**	**[3.87; 9.36]**
**Prediction interval**								**[-3.61; 16.84]**
Heterogeneity: $I^2 = 84\%$, $\tau^2 = 19.1011$, $p < 0.01$								
**HDI_class = High**								
Alizadeh et al. 2016	32	120.5	9.0	32	119.4	6.5	1.10	[-2.75; 4.95]
Falahi et al. 2011	70	123.2	9.9	78	120.9	7.9	2.30	[-0.61; 5.21]
Ouladsahebmadarek et al. 2011	410	134.6	7.5	372	124.8	9.1	9.80	[8.62; 10.98]
Ziaei et al. 2008	100	138.8	4.5	105	127.8	4.7	11.00	[9.74; 12.26]
Ziaei et al. 2007	370	137.5	10.5	357	125.6	12.4	11.90	[10.23; 13.57]
**Random effects model**	**982**			**944**			**7.44**	**[3.07; 11.81]**
**Prediction interval**								**[-9.50; 24.39]**
Heterogeneity: $I^2 = 93\%$, $\tau^2 = 23.3833$, $p < 0.01$								
**HDI_class = Low**								
Abioye et al. 2023	334	120.0	23.3	356	112.0	19.3	8.00	[4.80; 11.20]
**Prediction interval**								

Heterogeneity: $I^2 = 91\%$, $\tau^2 = 17.9753$, $p < 0.01$

Test for subgroup differences: $\chi^2_2 = 0.43$, df = 2 ($p = 0.81$)

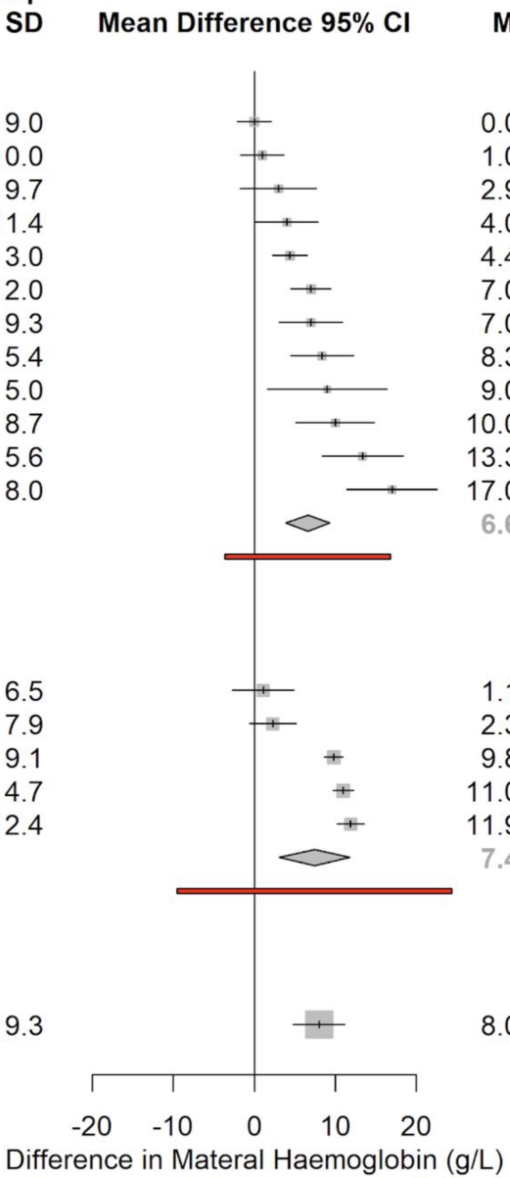

Figure S7 - Forest plot showing the effect of iron supplementation on maternal haemoglobin with subgroup analysis based on when the outcome was measured.

Study	Supplement Group Total	Supplement Group Mean	Supplement Group SD	Control Group Total	Control Group Mean	Control Group SD	MD	95%-CI
**When.Measured = At term**								
Alizadeh et al. 2016	32	120.5	9.0	32	119.4	6.5	1.10	[-2.75; 4.95]
Falahi et al. 2011	70	123.2	9.9	78	120.9	7.9	2.30	[-0.61; 5.21]
Buytaert et al. 1983	42	127.9	12.1	41	124.9	9.7	2.98	[-1.74; 7.70]
Makrides et al. 2003	200	127.0	13.0	193	120.0	12.0	7.00	[4.53; 9.47]
Milman 2023	44	126.0	8.4	36	119.0	9.3	7.00	[3.08; 10.92]
Abioye et al. 2023	334	120.0	23.3	356	112.0	19.3	8.00	[4.80; 11.20]
Ouladsahebmadarek et al. 2011	410	134.6	7.5	372	124.8	9.1	9.80	[8.62; 10.98]
Ziaei et al. 2008	100	138.8	4.5	105	127.8	4.7	11.00	[9.74; 12.26]
Ziaei et al. 2007	370	137.5	10.5	357	125.6	12.4	11.90	[10.23; 13.57]
**Random effects model**	**1602**			**1570**			**7.09**	**[4.54; 9.64]**
**Prediction interval**								**[-1.98; 16.16]**
Heterogeneity: $I^2 = 88\%$, $\tau^2 = 13.0163$, $p < 0.01$								
**When.Measured = After 34th week**								
Meier et al. 2003	48	121.7	6.7	44	117.7	11.4	4.01	[0.15; 7.87]
Tholin et al. 1995	25	126.0	7.6	19	117.6	5.4	8.39	[4.53; 12.25]
Harvey et al. 2007	6	126.0	8.0	7	117.0	5.0	9.00	[1.60; 16.40]
Svanberg et al. 1975	24	124.0	8.8	26	114.0	8.7	10.00	[5.14; 14.86]
Eskeland et al. 1997	24	126.7	11.0	20	113.4	5.6	13.36	[8.33; 18.38]
Puolakka et al. 1980	16	127.0	8.0	15	110.0	8.0	17.00	[11.36; 22.64]
**Random effects model**	**143**			**131**			**10.07**	**[6.38; 13.75]**
**Prediction interval**								**[-1.73; 21.86]**
Heterogeneity: $I^2 = 71\%$, $\tau^2 = 14.5084$, $p < 0.01$								
**When.Measured = Before 34th week**								
Siega-Riz et al. 2006	160	114.0	10.0	156	114.0	9.0	0.00	[-2.10; 2.10]
Cogswell et al. 2003	110	117.0	9.0	86	116.0	10.0	1.00	[-1.70; 3.70]
Parisi et al. 2017	45	112.4	4.7	12	108.0	3.0	4.40	[2.21; 6.59]
**Random effects model**	**315**			**254**			**1.82**	**[-0.87; 4.51]**
**Prediction interval**								**[-29.62; 33.25]**
Heterogeneity: $I^2 = 77\%$, $\tau^2 = 4.2377$, $p = 0.01$								
Heterogeneity: $I^2 = 91\%$, $\tau^2 = 17.9753$, $p < 0.01$								
Test for subgroup differences: $\chi^2_2 = 14.55$, df = 2 ($p < 0.01$)								

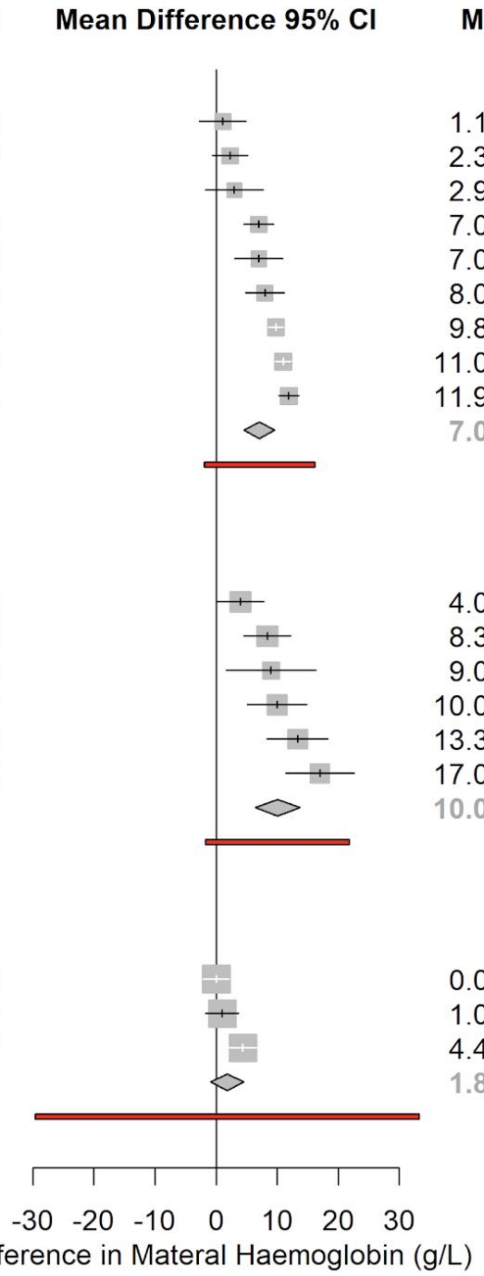

Figure S8 - Forest plot showing the effect of iron supplementation on maternal anaemia excluding studies with high risk of bias.

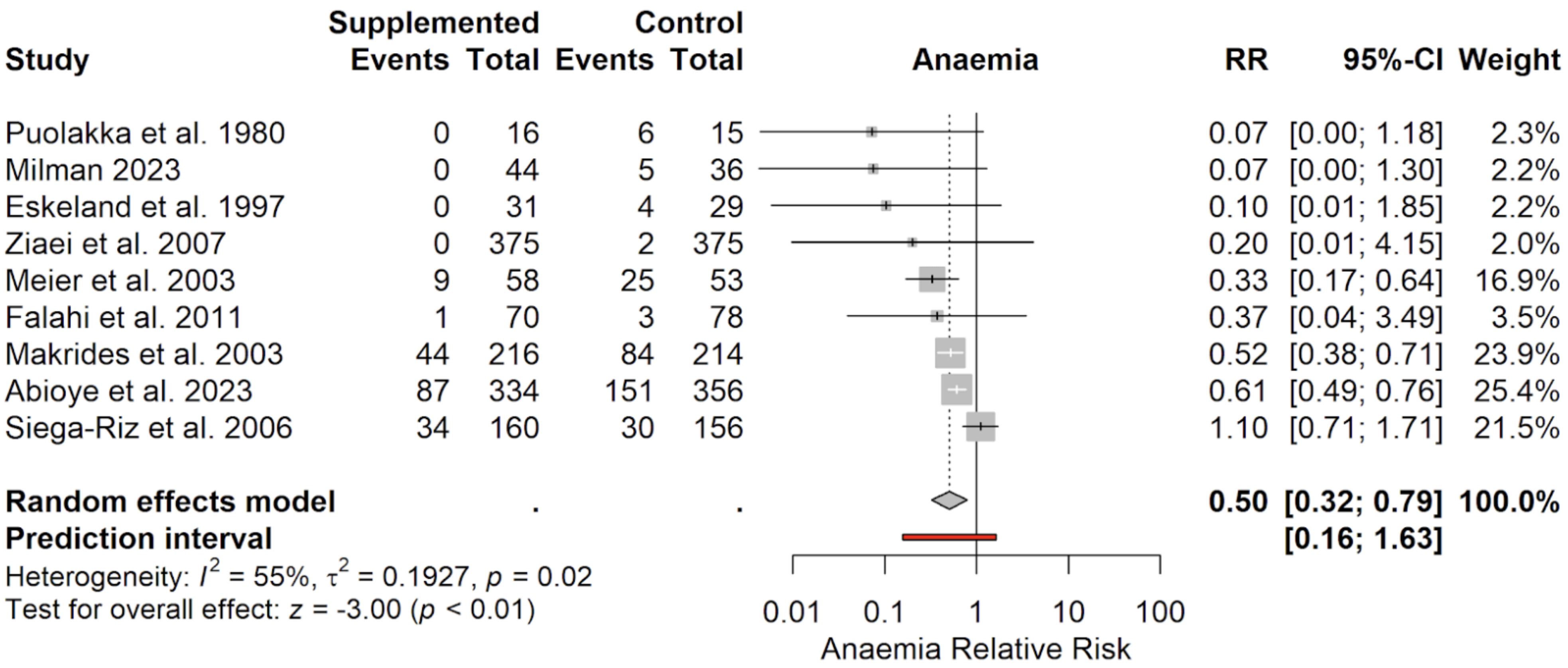

Figure S9 - Forest plot showing the effect of iron supplementation on maternal anaemia using Peto's method and odds ratio to avoid zero count cell corrections.

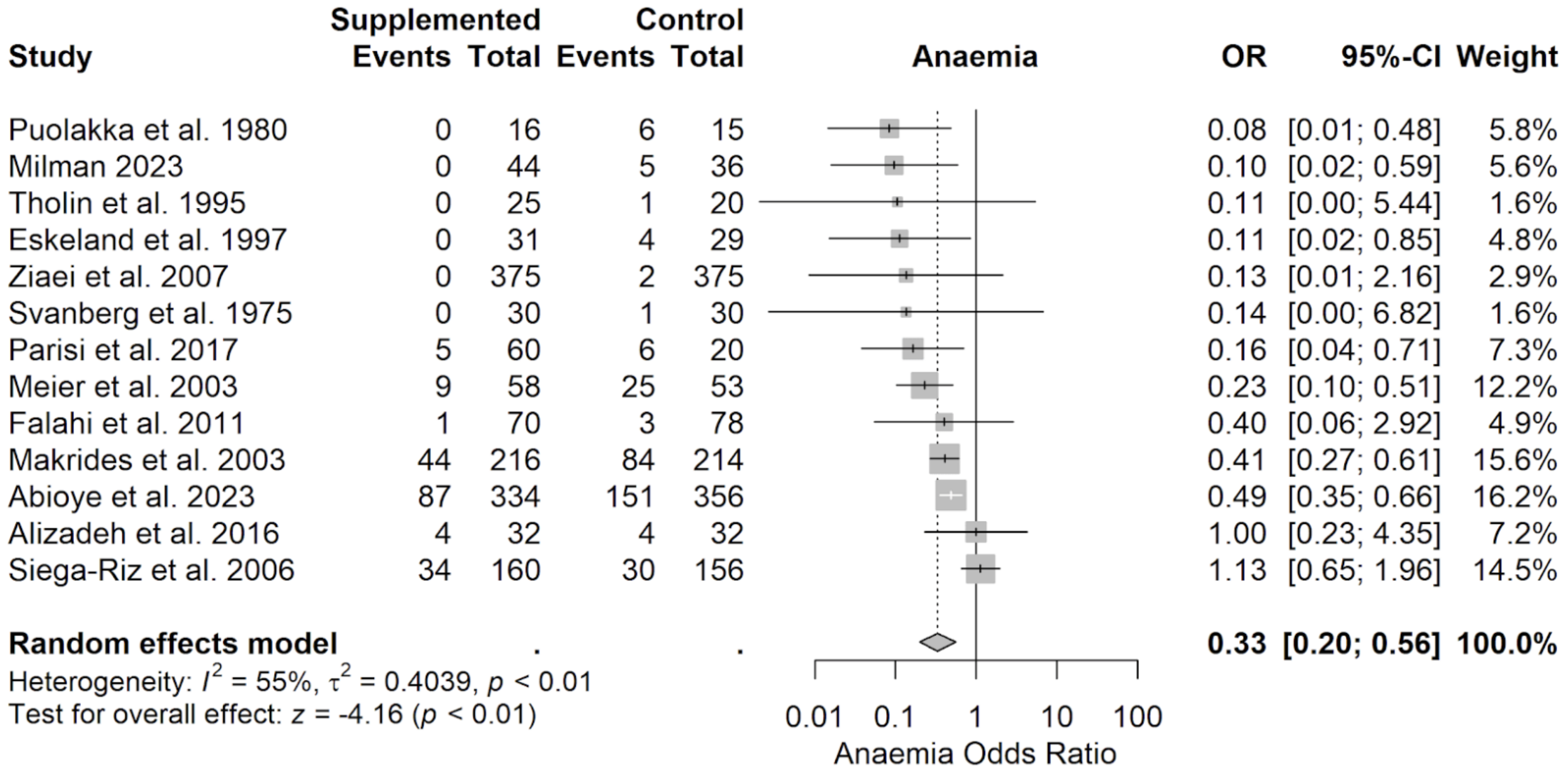

Figure S10 - Forest plot showing the effect of iron supplementation on maternal anaemia with subgroup analysis based on supplement dose.

Study	Supplemented Events	Supplemented Total	Control Events	Control Total	RR	95%-CI	Weight
Dose = Extreme							
Puolakka et al. 1980	0	16	6	15	0.07	[0.00; 1.18]	1.8%
Tholin et al. 1995	0	25	1	20	0.27	[0.01; 6.24]	1.4%
Svanberg et al. 1975	0	30	1	30	0.33	[0.01; 7.86]	1.4%
Random effects model		.		.	0.17	[0.03; 0.98]	4.5%
Prediction interval						[0.00; 13839.25]	
Heterogeneity: $I^2 = 0\%$, $\tau^2 = 0$, $p = 0.74$							
Dose = Low							
Eskeland et al. 1997	0	31	4	29	0.10	[0.01; 1.85]	1.7%
Parisi et al. 2017	5	60	6	20	0.28	[0.09; 0.81]	8.3%
Makrides et al. 2003	44	216	84	214	0.52	[0.38; 0.71]	20.2%
Siega-Riz et al. 2006	34	160	30	156	1.10	[0.71; 1.71]	17.8%
Random effects model		.		.	0.54	[0.27; 1.12]	47.9%
Prediction interval						[0.03; 10.20]	
Heterogeneity: $I^2 = 74\%$, $\tau^2 = 0.3297$, $p < 0.01$							
Dose = High							
Milman 2023	0	44	5	36	0.07	[0.00; 1.30]	1.7%
Ziaei et al. 2007	0	375	2	375	0.20	[0.01; 4.15]	1.5%
Meier et al. 2003	9	58	25	53	0.33	[0.17; 0.64]	13.7%
Falahi et al. 2011	1	70	3	78	0.37	[0.04; 3.49]	2.6%
Abioye et al. 2023	87	334	151	356	0.61	[0.49; 0.76]	21.6%
Alizadeh et al. 2016	4	32	4	32	1.00	[0.27; 3.66]	6.4%
Random effects model		.		.	0.50	[0.32; 0.78]	47.5%
Prediction interval						[0.18; 1.41]	
Heterogeneity: $I^2 = 21\%$, $\tau^2 = 0.0865$, $p = 0.28$							
**Random effects model**		**.**		**.**	**0.50**	**[0.34; 0.74]**	**100.0%**
**Prediction interval**						**[0.19; 1.36]**	

Heterogeneity: $I^2 = 42\%$, $\tau^2 = 0.1657$, $p = 0.05$
Test for overall effect: $z = -3.51$ ($p < 0.01$)
Test for subgroup differences: $\chi^2_2 = 1.47$, df = 2 ($p = 0.48$)

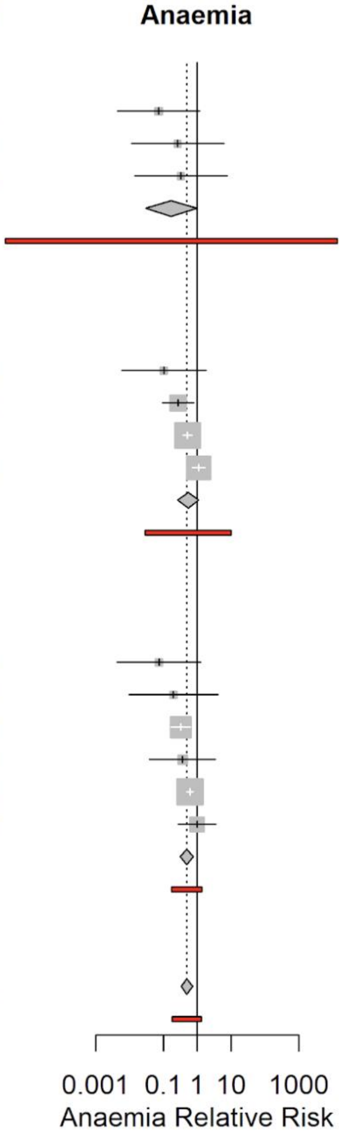

Figure S11 - Forest plot showing the effect of iron supplementation on maternal anaemia with subgroup analysis based on the study's definition of anaemia in their inclusion criteria. As compared to the WHO definition of less than 110g/L.

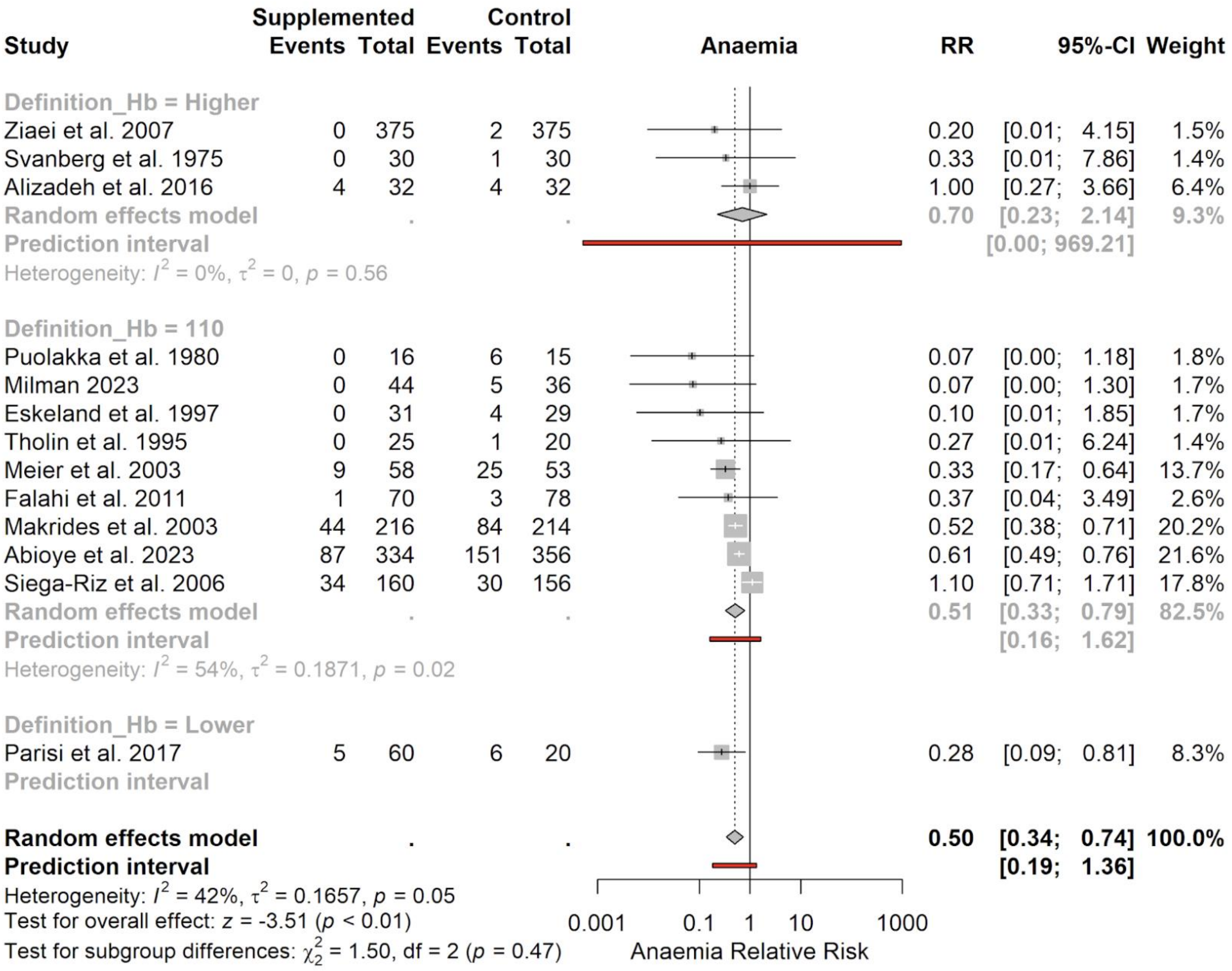

Figure S12 - Forest plot showing the effect of iron supplementation on maternal anaemia with subgroup analysis based on when during pregnancy supplements were started.

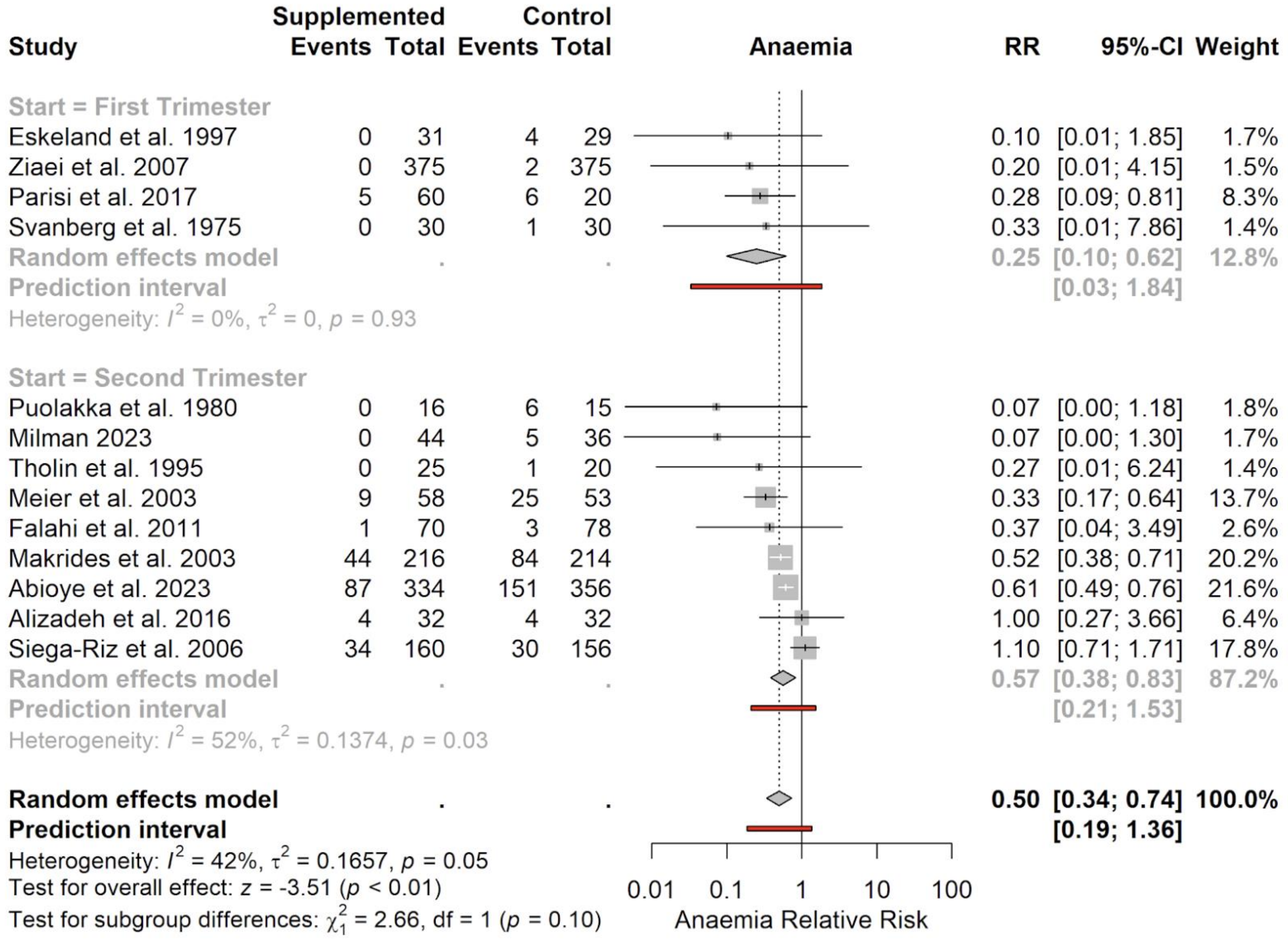

Figure S13 - Forest plot showing the effect of iron supplementation on maternal anaemia with subgroup analysis based on human development index in the country and year of the study.

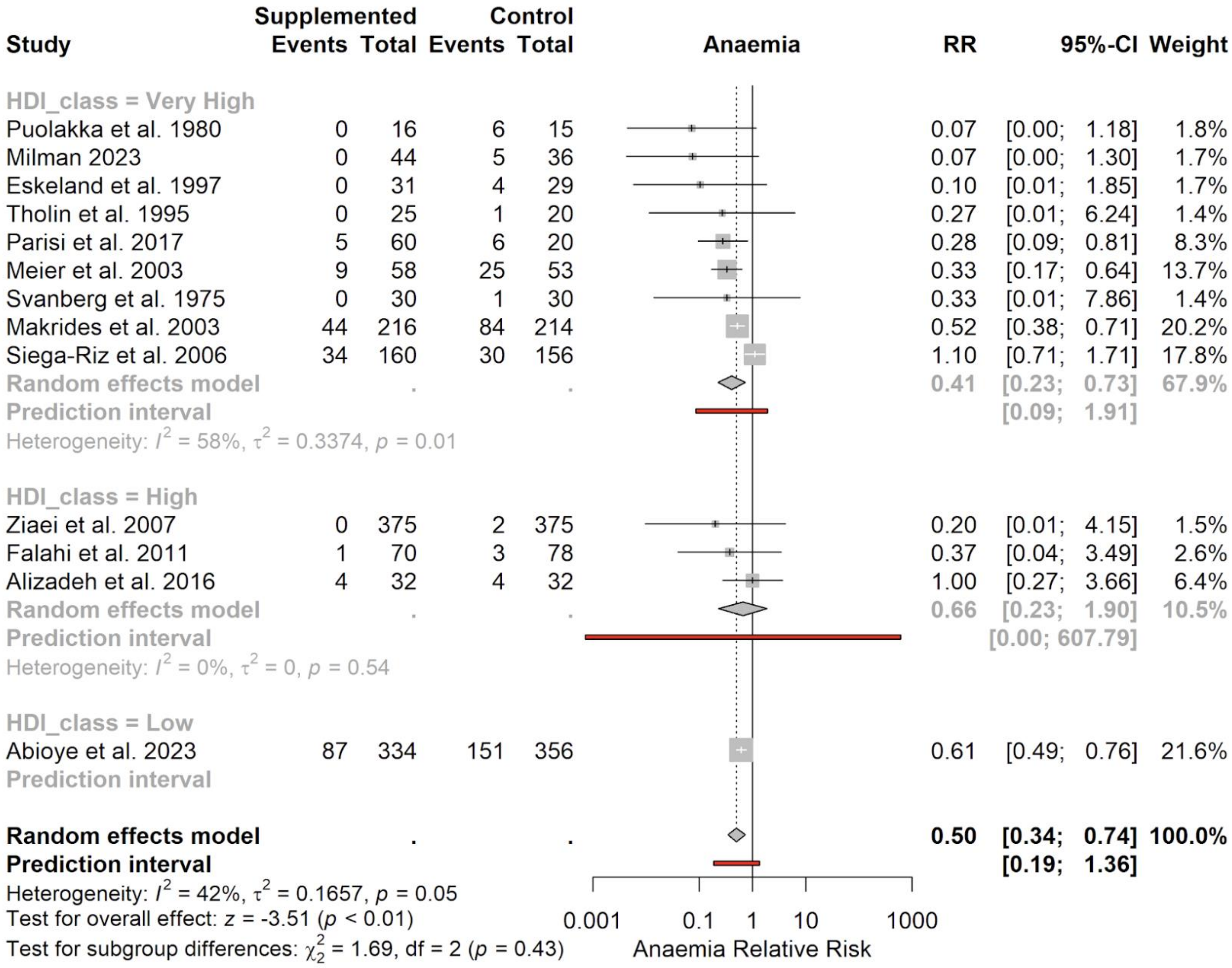

Figure S14 - Funnel plot of studies reporting the effect of iron supplementation on risk of maternal anaemia.

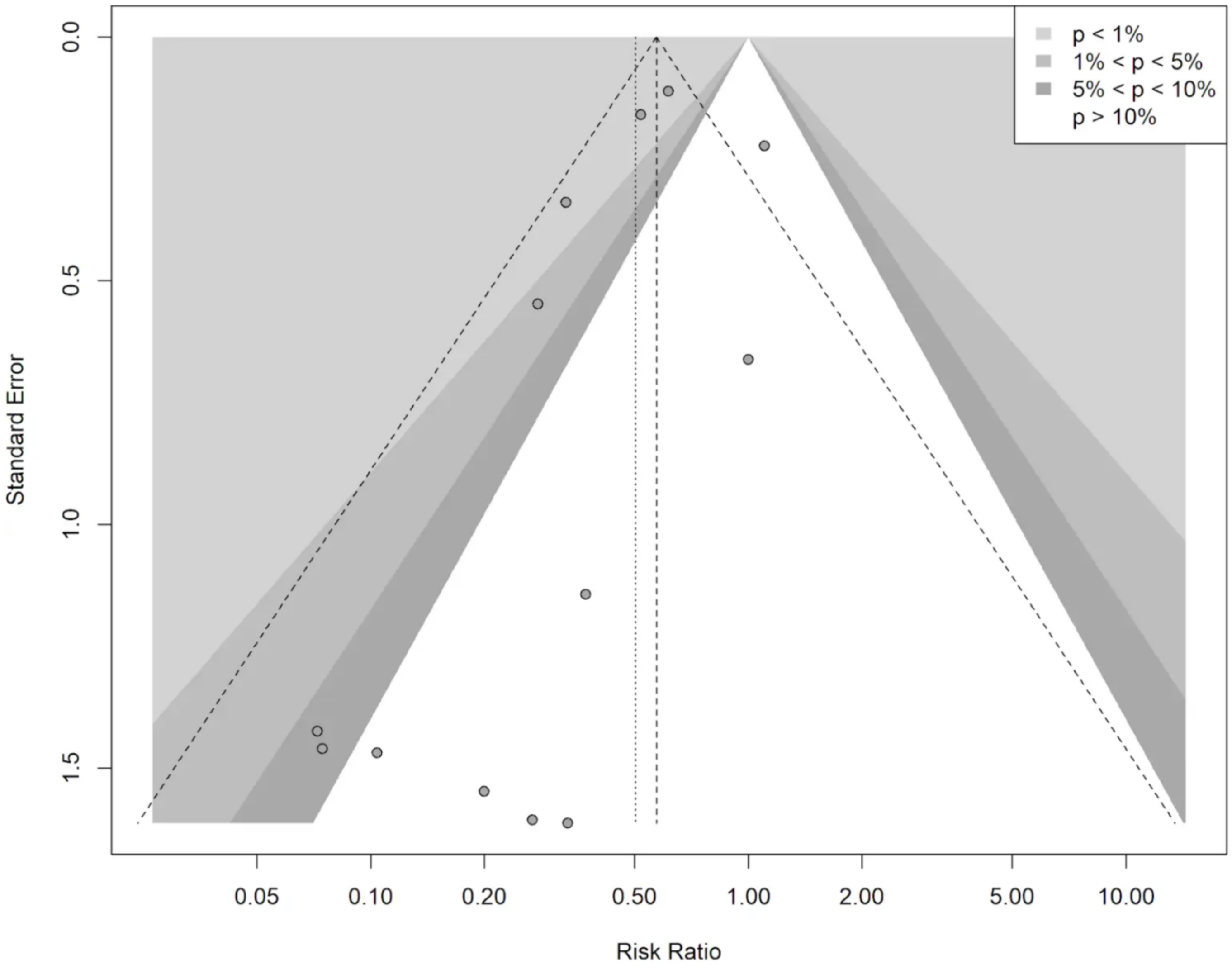

Figure S15 - Forest plot showing effect of iron supplementation on maternal anaemia excluding studies with fewer than 100 participants.

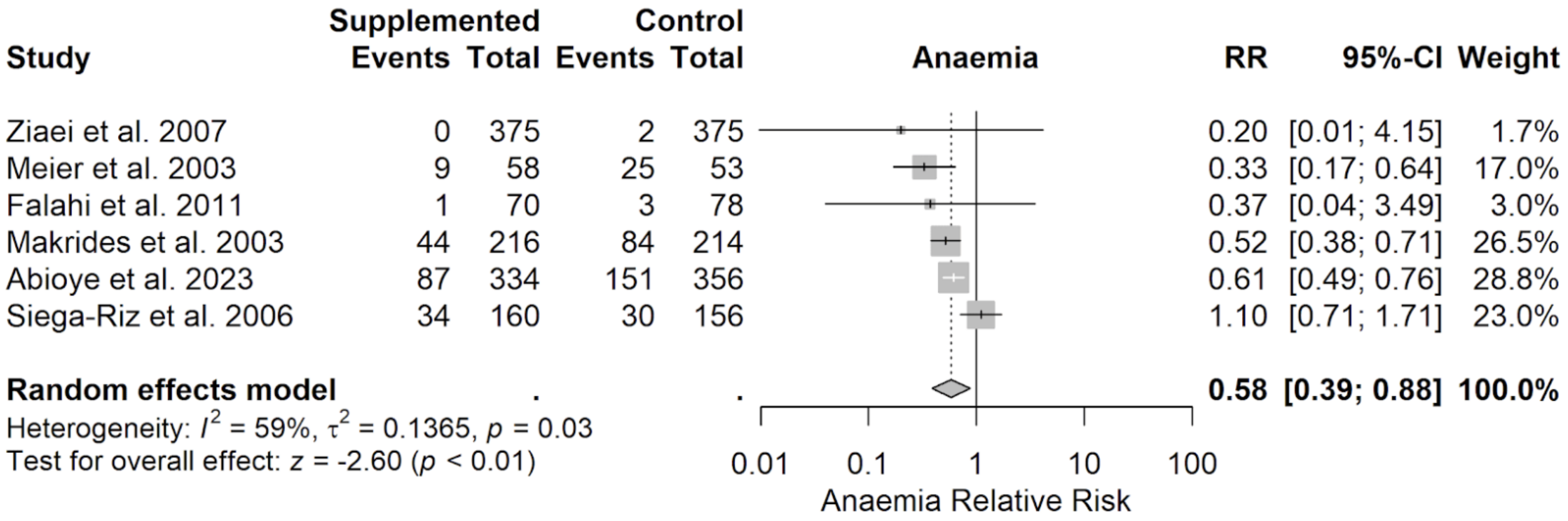

Figure S16 - Forest plot showing the effect of iron supplementation on maternal ferritin.

Study	Supplement Group Total	Supplement Group Mean	Supplement Group SD	Control Group Total	Control Group Mean	Control Group SD	Mean Difference 95% CI	MD	95%-CI	Weight
Alizadeh et al. 2016	32	28.5	9.3	32	27.2	13.0		1.28	[-4.25; 6.81]	7.6%
Siega-Riz et al. 2006	160	22.2	15.5	156	20.3	19.8		1.90	[-2.03; 5.83]	8.0%
Tholin et al. 1995	25	19.2	8.4	19	13.6	12.2		5.54	[-0.86; 11.93]	7.4%
Falahi et al. 2011	70	29.1	16.7	78	23.0	17.7		6.10	[0.56; 11.64]	7.6%
Makrides et al. 2003	186	21.0	18.0	176	14.0	10.0		7.00	[4.02; 9.98]	8.1%
Ziaei et al. 2008	100	26.2	11.2	105	19.1	10.7		7.10	[4.10; 10.10]	8.1%
Parisi et al. 2017	45	34.0	21.2	12	26.1	16.5		7.85	[-3.36; 19.06]	6.1%
Eskeland et al. 1997	24	18.7	12.6	20	8.6	4.0		10.08	[4.76; 15.39]	7.7%
Harvey et al. 2007	6	19.8	11.5	7	7.3	4.8		12.50	[2.64; 22.36]	6.5%
Meier et al. 2003	33	26.3	32.9	26	10.8	8.6		15.52	[3.82; 27.22]	5.9%
Ouladsahebmadarek et al. 2011	410	26.9	42.7	372	9.3	12.0		17.65	[13.34; 21.96]	7.9%
Buytaert et al. 1983	18	45.2	22.9	20	23.9	9.9		21.30	[9.87; 32.73]	6.0%
Abioye et al. 2023	334	67.2	54.1	356	42.0	41.4		25.20	[17.98; 32.42]	7.2%
Puolakka et al. 1980	16	63.0	19.0	15	21.0	14.0		42.00	[30.30; 53.70]	5.9%
**Random effects model**	.			.				**12.22**	**[6.92; 17.52]**	**100.0%**
**Prediction interval**									**[-9.05; 33.49]**	

Heterogeneity: $I^2 = 87\%$, $\tau^2 = 87.9517$, $p < 0.01$

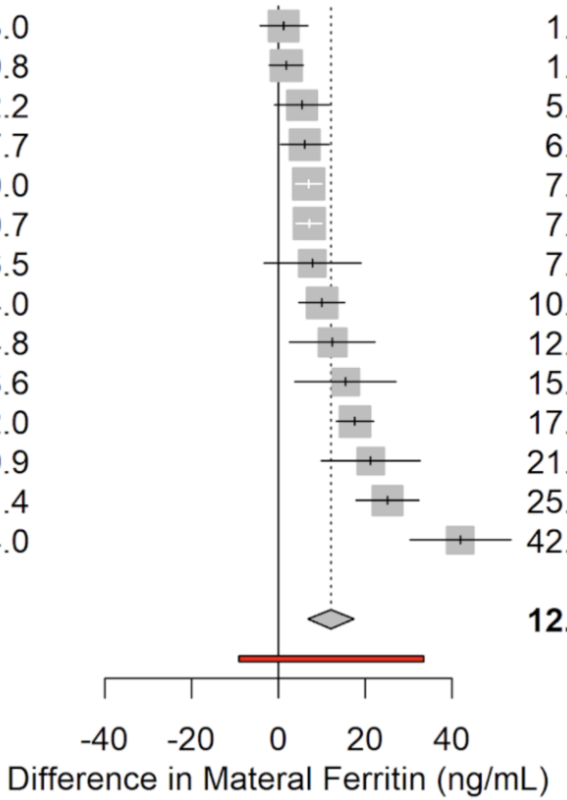

Figure S17 **-** *Forest plot showing the effect of iron supplementation on maternal ferritin excluding studies with a high risk of bias.*

Study	Supplement Group Total	Supplement Group Mean	Supplement Group SD	Control Group Total	Control Group Mean	Control Group SD	MD	95%-CI	Weight
Siega-Riz et al. 2006	160	22.2	15.5	156	20.3	19.8	1.90	[-2.03; 5.83]	12.3%
Falahi et al. 2011	70	29.1	16.7	78	23.0	17.7	6.10	[0.56; 11.64]	11.9%
Makrides et al. 2003	186	21.0	18.0	176	14.0	10.0	7.00	[4.02; 9.98]	12.4%
Eskeland et al. 1997	24	18.7	12.6	20	8.6	4.0	10.08	[4.76; 15.39]	11.9%
Harvey et al. 2007	6	19.8	11.5	7	7.3	4.8	12.50	[2.64; 22.36]	10.5%
Meier et al. 2003	33	26.3	32.9	26	10.8	8.6	15.52	[3.82; 27.22]	9.8%
Buytaert et al. 1983	18	45.2	22.9	20	23.9	9.9	21.30	[9.87; 32.73]	9.9%
Abioye et al. 2023	334	67.2	54.1	356	42.0	41.4	25.20	[17.98; 32.42]	11.4%
Puolakka et al. 1980	16	63.0	19.0	15	21.0	14.0	42.00	[30.30; 53.70]	9.8%
**Random effects model**	**.**			**.**			**14.98**	**[7.23; 22.73]**	**100.0%**
**Prediction interval**								**[-12.91; 42.88]**	

Heterogeneity: $I^2 = 89\%$, $\tau^2 = 123.5035$, $p < 0.01$

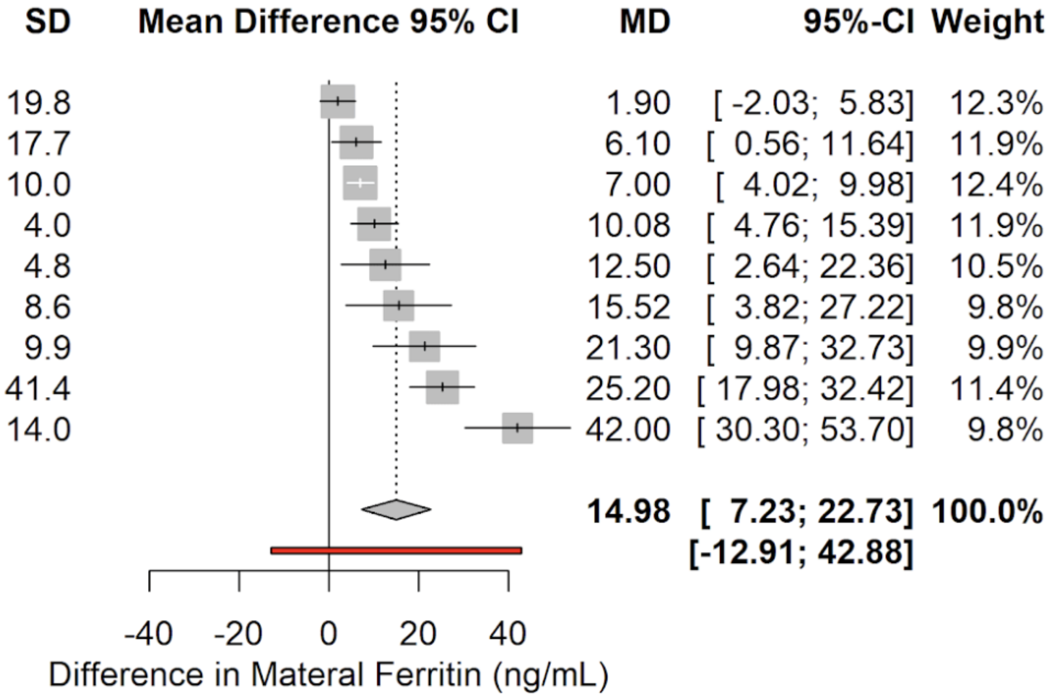

Figure S18 - Forest plot showing the effect of iron supplementation on maternal ferritin with subgroup analysis based on dose of iron supplement.

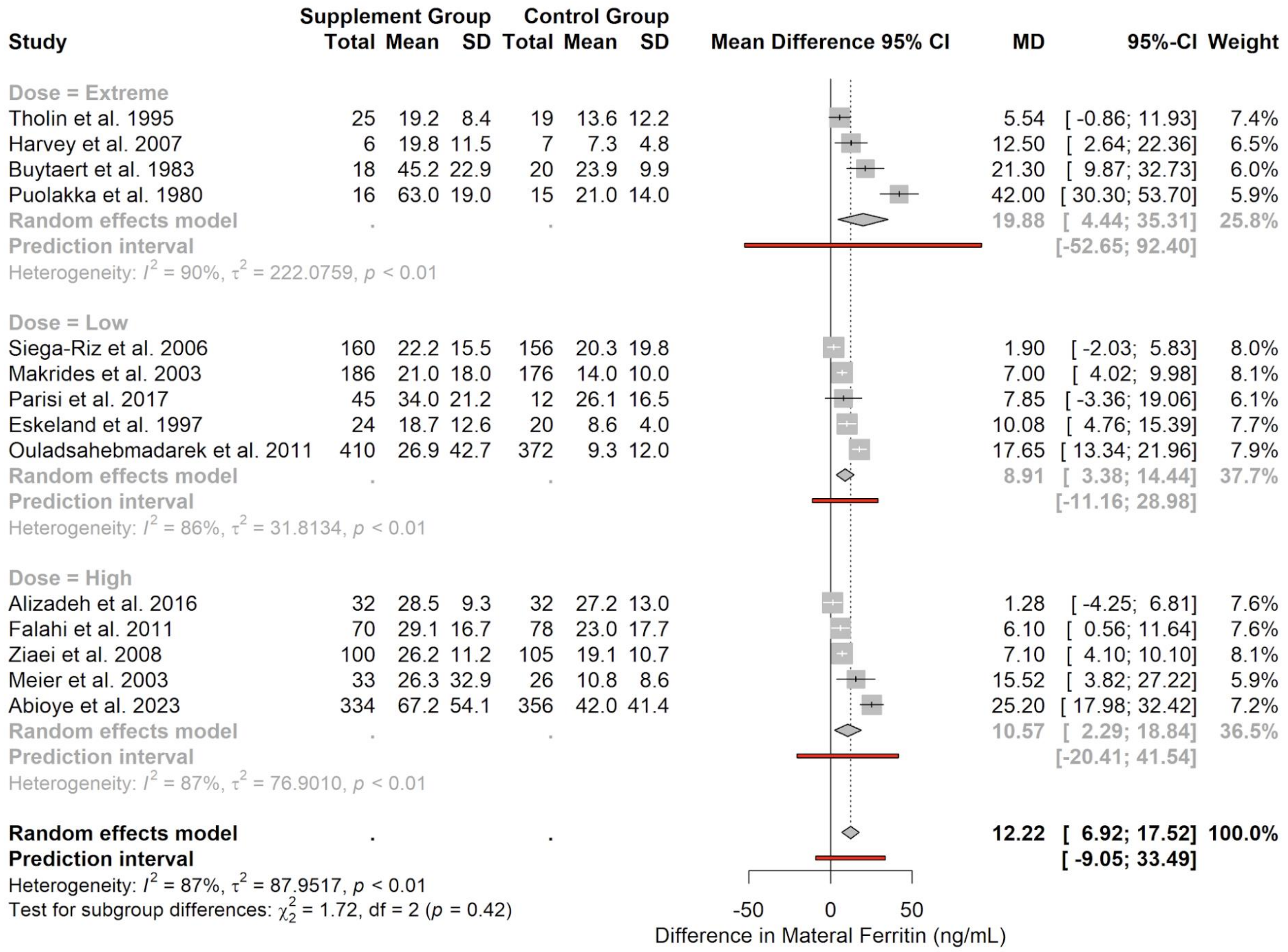

Figure S19 - Forest plot showing the effect of iron supplementation on maternal ferritin with subgroup analysis based on the study's definition of anaemia in their inclusion criteria. As compared to the WHO definition of less than 110g/L.

Study	Supplement Group Total	Supplement Group Mean	Supplement Group SD	Control Group Total	Control Group Mean	Control Group SD	MD	95%-CI	Weight
**Definition_Hb = 110**									
Siega-Riz et al. 2006	160	22.2	15.5	156	20.3	19.8	1.90	[-2.03; 5.83]	8.0%
Tholin et al. 1995	25	19.2	8.4	19	13.6	12.2	5.54	[-0.86; 11.93]	7.4%
Falahi et al. 2011	70	29.1	16.7	78	23.0	17.7	6.10	[0.56; 11.64]	7.6%
Makrides et al. 2003	186	21.0	18.0	176	14.0	10.0	7.00	[4.02; 9.98]	8.1%
Eskeland et al. 1997	24	18.7	12.6	20	8.6	4.0	10.08	[4.76; 15.39]	7.7%
Meier et al. 2003	33	26.3	32.9	26	10.8	8.6	15.52	[3.82; 27.22]	5.9%
Abioye et al. 2023	334	67.2	54.1	356	42.0	41.4	25.20	[17.98; 32.42]	7.2%
Puolakka et al. 1980	16	63.0	19.0	15	21.0	14.0	42.00	[30.30; 53.70]	5.9%
Random effects model	.			.			13.47	[4.76; 22.19]	57.8%
Prediction interval								[-17.88; 44.83]	
Heterogeneity: $I^2 = 90\%$, $\tau^2 = 144.4057$, $p < 0.01$									
**Definition_Hb = Higher**									
Alizadeh et al. 2016	32	28.5	9.3	32	27.2	13.0	1.28	[-4.25; 6.81]	7.6%
Ziaei et al. 2008	100	26.2	11.2	105	19.1	10.7	7.10	[4.10; 10.10]	8.1%
Ouladsahebmadarek et al. 2011	410	26.9	42.7	372	9.3	12.0	17.65	[13.34; 21.96]	7.9%
Buytaert et al. 1983	18	45.2	22.9	20	23.9	9.9	21.30	[9.87; 32.73]	6.0%
Random effects model	.			.			11.18	[2.47; 19.88]	29.6%
Prediction interval								[-29.19; 51.54]	
Heterogeneity: $I^2 = 90\%$, $\tau^2 = 68.2809$, $p < 0.01$									
**Definition_Hb = Lower**									
Parisi et al. 2017	45	34.0	21.2	12	26.1	16.5	7.85	[-3.36; 19.06]	6.1%
Harvey et al. 2007	6	19.8	11.5	7	7.3	4.8	12.50	[2.64; 22.36]	6.5%
Random effects model	.			.			10.47	[3.07; 17.88]	12.5%
Prediction interval									
Heterogeneity: $I^2 = 0\%$, $\tau^2 = 0$, $p = 0.54$									
**Random effects model**	**.**			**.**			**12.22**	**[6.92; 17.52]**	**100.0%**
**Prediction interval**								**[-9.05; 33.49]**	
Heterogeneity: $I^2 = 87\%$, $\tau^2 = 87.9517$, $p < 0.01$									
Test for subgroup differences: $\chi^2_2 = 0.28$, df = 2 ($p = 0.87$)									

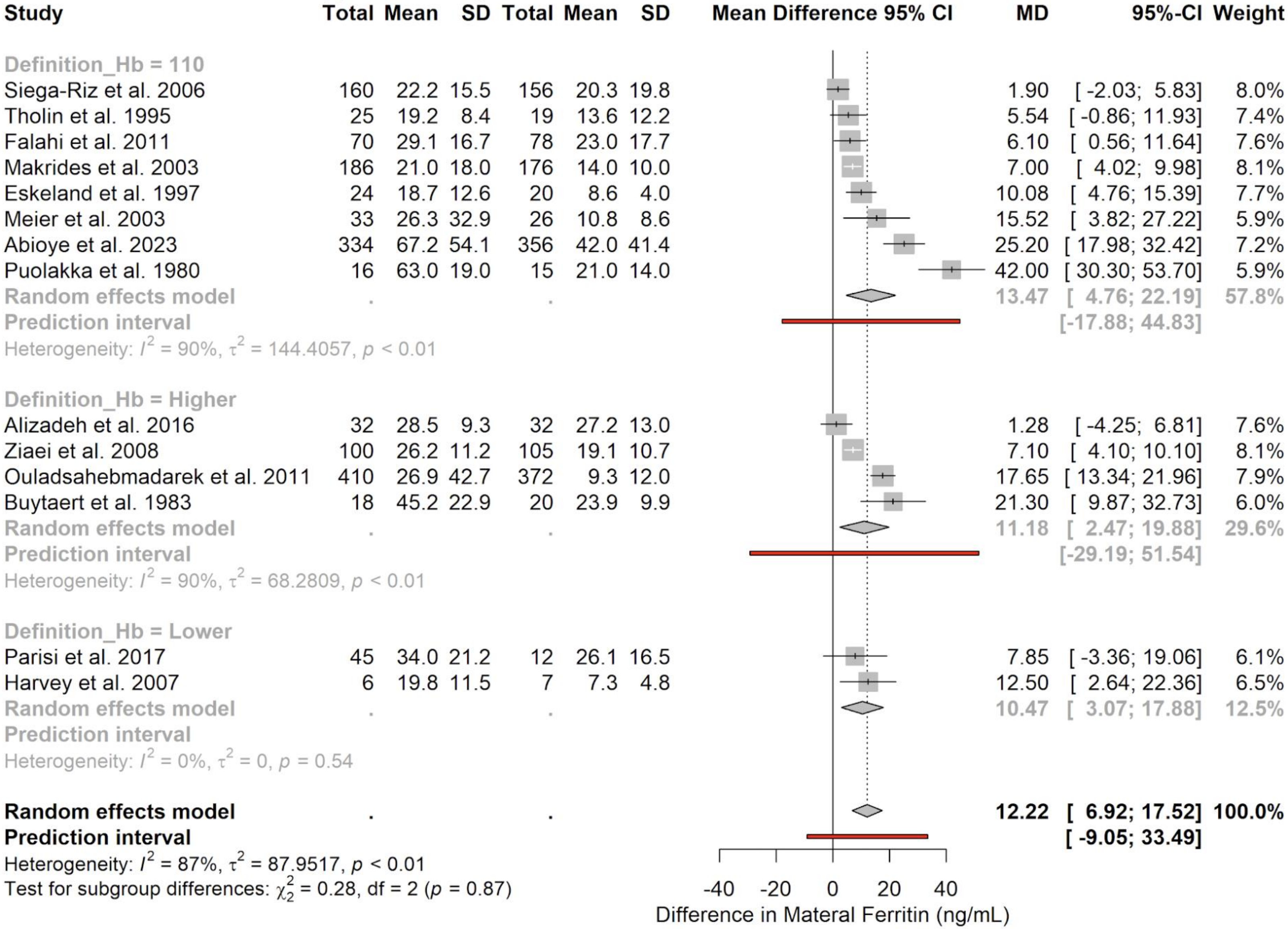

Figure S20 - Forest plot showing the effect of iron supplementation on maternal ferritin with subgroup analysis based on when during pregnancy iron supplements were started.

Study	Supplement Group Total	Supplement Group Mean	Supplement Group SD	Control Group Total	Control Group Mean	Control Group SD	MD	95%-CI	Weight
**Start = Second Trimester**									
Alizadeh et al. 2016	32	28.5	9.3	32	27.2	13.0	1.28	[-4.25; 6.81]	7.6%
Siega-Riz et al. 2006	160	22.2	15.5	156	20.3	19.8	1.90	[-2.03; 5.83]	8.0%
Tholin et al. 1995	25	19.2	8.4	19	13.6	12.2	5.54	[-0.86; 11.93]	7.4%
Falahi et al. 2011	70	29.1	16.7	78	23.0	17.7	6.10	[0.56; 11.64]	7.6%
Makrides et al. 2003	186	21.0	18.0	176	14.0	10.0	7.00	[4.02; 9.98]	8.1%
Ziaei et al. 2008	100	26.2	11.2	105	19.1	10.7	7.10	[4.10; 10.10]	8.1%
Harvey et al. 2007	6	19.8	11.5	7	7.3	4.8	12.50	[2.64; 22.36]	6.5%
Meier et al. 2003	33	26.3	32.9	26	10.8	8.6	15.52	[3.82; 27.22]	5.9%
Buytaert et al. 1983	18	45.2	22.9	20	23.9	9.9	21.30	[9.87; 32.73]	6.0%
Abioye et al. 2023	334	67.2	54.1	356	42.0	41.4	25.20	[17.98; 32.42]	7.2%
Puolakka et al. 1980	16	63.0	19.0	15	21.0	14.0	42.00	[30.30; 53.70]	5.9%
**Random effects model**	.			.			12.43	[5.62; 19.25]	78.4%
**Prediction interval**								[-13.35; 38.21]	
Heterogeneity: $I^2 = 87\%$, $\tau^2 = 117.7917$, $p < 0.01$									
**Start = First Trimester**									
Parisi et al. 2017	45	34.0	21.2	12	26.1	16.5	7.85	[-3.36; 19.06]	6.1%
Eskeland et al. 1997	24	18.7	12.6	20	8.6	4.0	10.08	[4.76; 15.39]	7.7%
Ouladsahebmadarek et al. 2011	410	26.9	42.7	372	9.3	12.0	17.65	[13.34; 21.96]	7.9%
**Random effects model**	.			.			12.87	[6.75; 18.99]	21.6%
**Prediction interval**								[-54.21; 79.95]	
Heterogeneity: $I^2 = 67\%$, $\tau^2 = 18.1173$, $p = 0.05$									
**Random effects model**	.			.			**12.22**	**[6.92; 17.52]**	**100.0%**
**Prediction interval**								**[-9.05; 33.49]**	

Heterogeneity: $I^2 = 87\%$, $\tau^2 = 87.9517$, $p < 0.01$
Test for subgroup differences: $\chi^2_1 = 0.01$, df = 1 ($p = 0.93$)

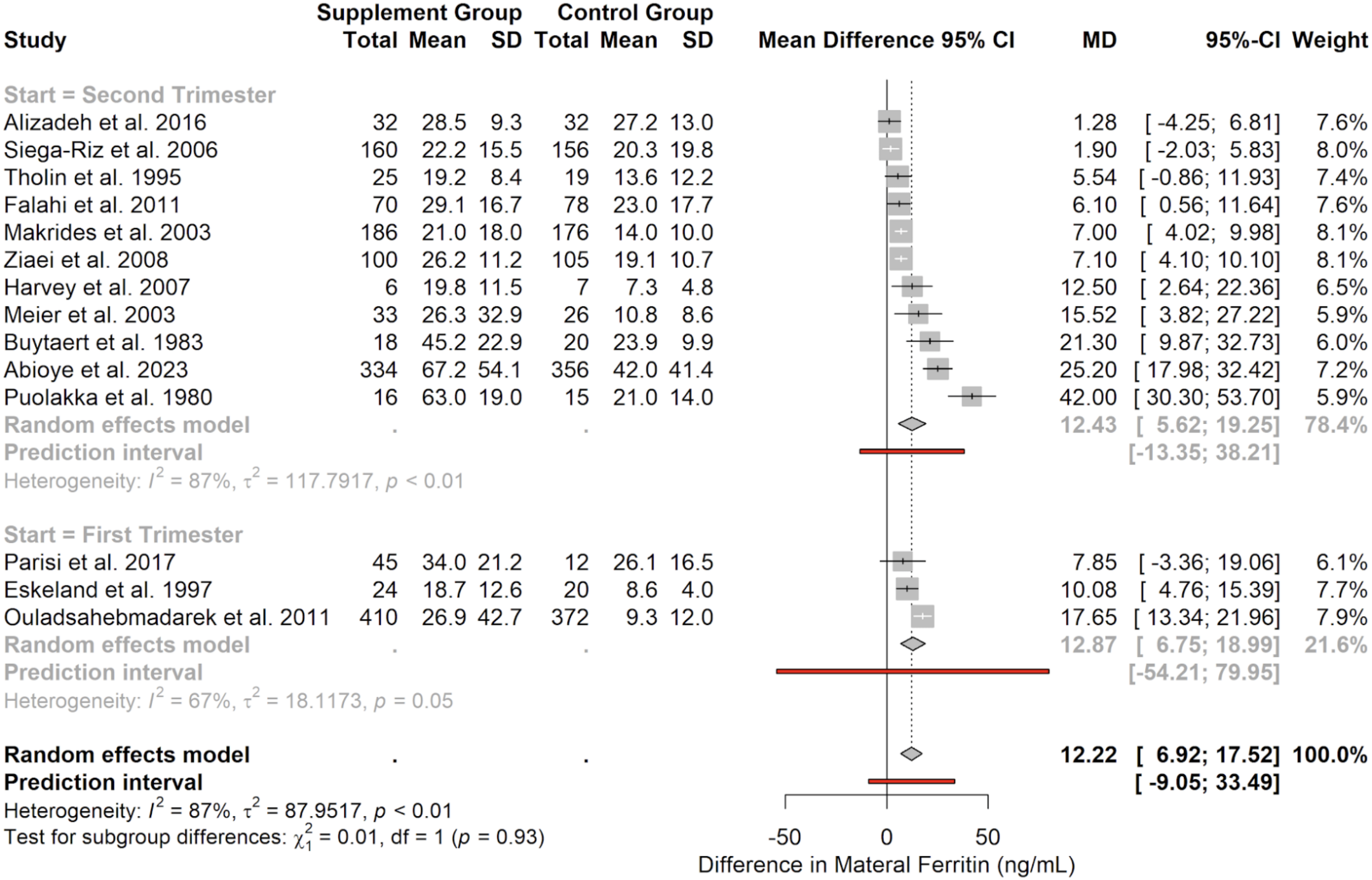

Figure S21 - Forest plot showing the effect of iron supplementation on maternal ferritin with subgroup analysis based on when the outcome was measured.

Study	Supplement Group Total	Supplement Group Mean	Supplement Group SD	Control Group Total	Control Group Mean	Control Group SD	MD	95%-CI	Weight
**When.Measured = After 34th week**									
Tholin et al. 1995	25	19.2	8.4	19	13.6	12.2	5.54	[-0.86; 11.93]	7.4%
Eskeland et al. 1997	24	18.7	12.6	20	8.6	4.0	10.08	[4.76; 15.39]	7.7%
Harvey et al. 2007	6	19.8	11.5	7	7.3	4.8	12.50	[2.64; 22.36]	6.5%
Meier et al. 2003	33	26.3	32.9	26	10.8	8.6	15.52	[3.82; 27.22]	5.9%
Puolakka et al. 1980	16	63.0	19.0	15	21.0	14.0	42.00	[30.30; 53.70]	5.9%
**Random effects model**	.			.			16.57	[4.41; 28.73]	33.4%
**Prediction interval**								[-29.41; 62.56]	
Heterogeneity: $I^2 = 87\%$, $\tau^2 = 170.2970$, $p < 0.01$									
**When.Measured = At term**									
Alizadeh et al. 2016	32	28.5	9.3	32	27.2	13.0	1.28	[-4.25; 6.81]	7.6%
Falahi et al. 2011	70	29.1	16.7	78	23.0	17.7	6.10	[0.56; 11.64]	7.6%
Makrides et al. 2003	186	21.0	18.0	176	14.0	10.0	7.00	[4.02; 9.98]	8.1%
Ziaei et al. 2008	100	26.2	11.2	105	19.1	10.7	7.10	[4.10; 10.10]	8.1%
Ouladsahebmadarek et al. 2011	410	26.9	42.7	372	9.3	12.0	17.65	[13.34; 21.96]	7.9%
Buytaert et al. 1983	18	45.2	22.9	20	23.9	9.9	21.30	[9.87; 32.73]	6.0%
Abioye et al. 2023	334	67.2	54.1	356	42.0	41.4	25.20	[17.98; 32.42]	7.2%
**Random effects model**	.			.			11.67	[5.23; 18.11]	52.6%
**Prediction interval**								[-10.91; 34.24]	
Heterogeneity: $I^2 = 88\%$, $\tau^2 = 66.3195$, $p < 0.01$									
**When.Measured = Before 34th week**									
Siega-Riz et al. 2006	160	22.2	15.5	156	20.3	19.8	1.90	[-2.03; 5.83]	8.0%
Parisi et al. 2017	45	34.0	21.2	12	26.1	16.5	7.85	[-3.36; 19.06]	6.1%
**Random effects model**	.			.			2.55	[-1.16; 6.26]	14.0%
**Prediction interval**									
Heterogeneity: $I^2 = 0\%$, $\tau^2 = 0$, $p = 0.33$									
**Random effects model**	.			.			**12.22**	**[6.92; 17.52]**	**100.0%**
**Prediction interval**								**[-9.05; 33.49]**	
Heterogeneity: $I^2 = 87\%$, $\tau^2 = 87.9517$, $p < 0.01$									
Test for subgroup differences: $\chi^2_2 = 9.14$, df = 2 ($p = 0.01$)									

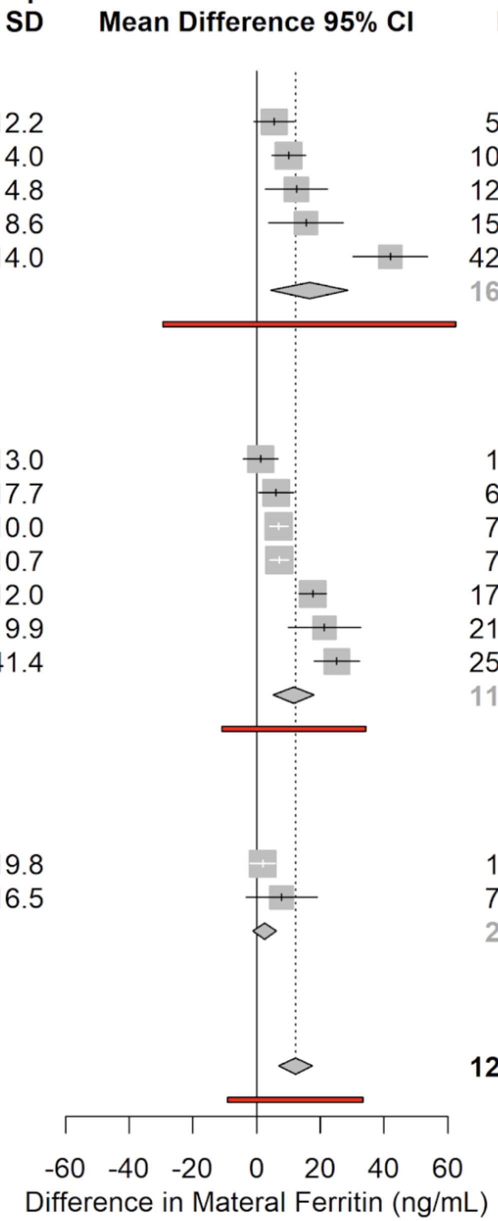

Figure S22 - Forest plot showing the effect of iron supplementation on maternal ferritin with subgroup analysis based on human development index in the country and year of the study.

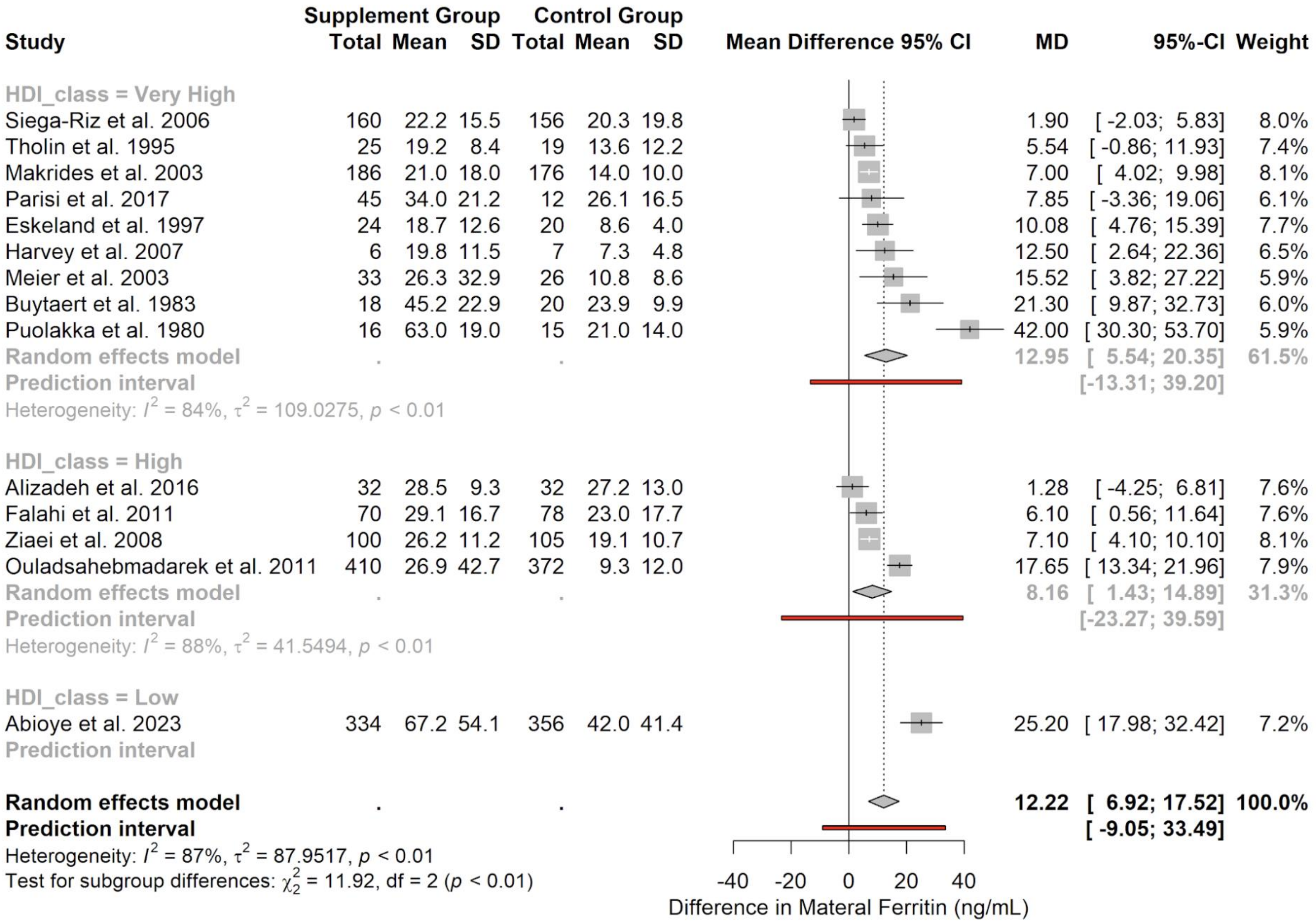

Figure S23 - Forest plot showing the effect of iron supplementation on birthweight.

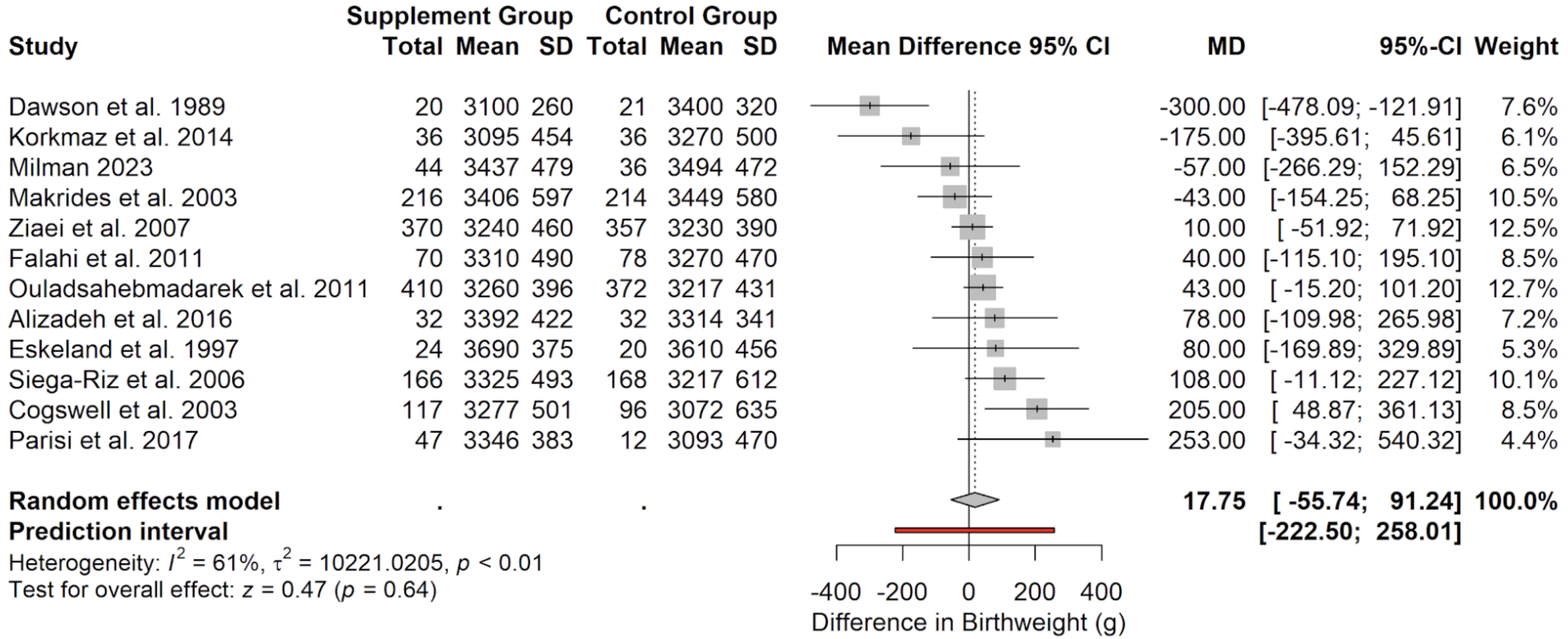

Figure S24 - Forest plot showing the effect of iron supplementation on birthweight with subgroup analysis based on dose of iron supplement.

Study	Supplement Group Total	Supplement Group Mean	Supplement Group SD	Control Group Total	Control Group Mean	Control Group SD	MD	95%-CI	Weight
**Dose = Low**									
Dawson et al. 1989	20	3100	260	21	3400	320	-300.00	[-478.09; -121.91]	7.6%
Makrides et al. 2003	216	3406	597	214	3449	580	-43.00	[-154.25; 68.25]	10.5%
Ouladsahebmadarek et al. 2011	410	3260	396	372	3217	431	43.00	[-15.20; 101.20]	12.7%
Eskeland et al. 1997	24	3690	375	20	3610	456	80.00	[-169.89; 329.89]	5.3%
Siega-Riz et al. 2006	166	3325	493	168	3217	612	108.00	[-11.12; 227.12]	10.1%
Cogswell et al. 2003	117	3277	501	96	3072	635	205.00	[48.87; 361.13]	8.5%
Parisi et al. 2017	47	3346	383	12	3093	470	253.00	[-34.32; 540.32]	4.4%
**Random effects model**	.			.			**40.03**	**[-86.20; 166.25]**	**59.1%**
**Prediction interval**								**[-374.36; 454.41]**	
Heterogeneity: $I^2 = 75\%$, $\tau^2 = 21839.4976$, $p < 0.01$									
**Dose = High**									
Korkmaz et al. 2014	36	3095	454	36	3270	500	-175.00	[-395.61; 45.61]	6.1%
Milman 2023	44	3437	479	36	3494	472	-57.00	[-266.29; 152.29]	6.5%
Ziaei et al. 2007	370	3240	460	357	3230	390	10.00	[-51.92; 71.92]	12.5%
Falahi et al. 2011	70	3310	490	78	3270	470	40.00	[-115.10; 195.10]	8.5%
Alizadeh et al. 2016	32	3392	422	32	3314	341	78.00	[-109.98; 265.98]	7.2%
**Random effects model**	.			.			**4.23**	**[-47.47; 55.93]**	**40.9%**
**Prediction interval**								**[-79.72; 88.18]**	
Heterogeneity: $I^2 = 0\%$, $\tau^2 = < 0.0001$, $p = 0.45$									
**Random effects model**	.			.			**17.75**	**[-55.74; 91.24]**	**100.0%**
**Prediction interval**								**[-222.50; 258.01]**	

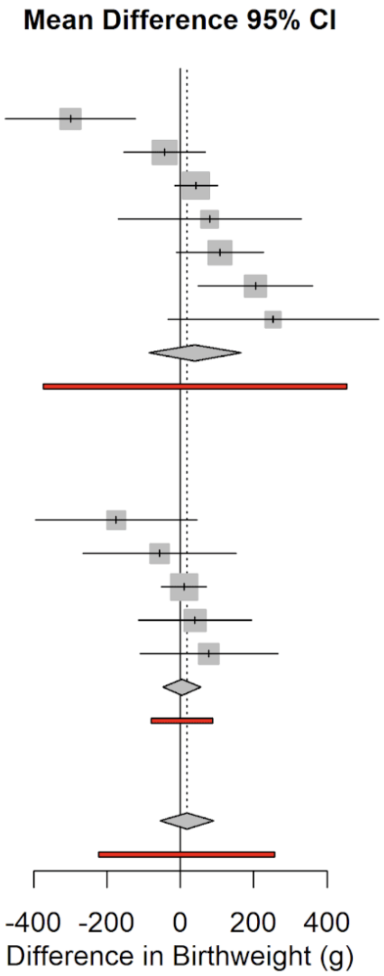

Heterogeneity: $I^2 = 61\%$, $\tau^2 = 10221.0205$, $p < 0.01$
Test for overall effect: $z = 0.47$ ($p = 0.64$)
Test for subgroup differences: $\chi^2_1 = 0.26$, df = 1 ($p = 0.61$)

Figure S25 - Forest plot showing the effect of iron supplementation on birthweight with subgroup analysis based on the study's definition of anaemia in their inclusion criteria. As compared to the WHO definition of less than 110g/L.

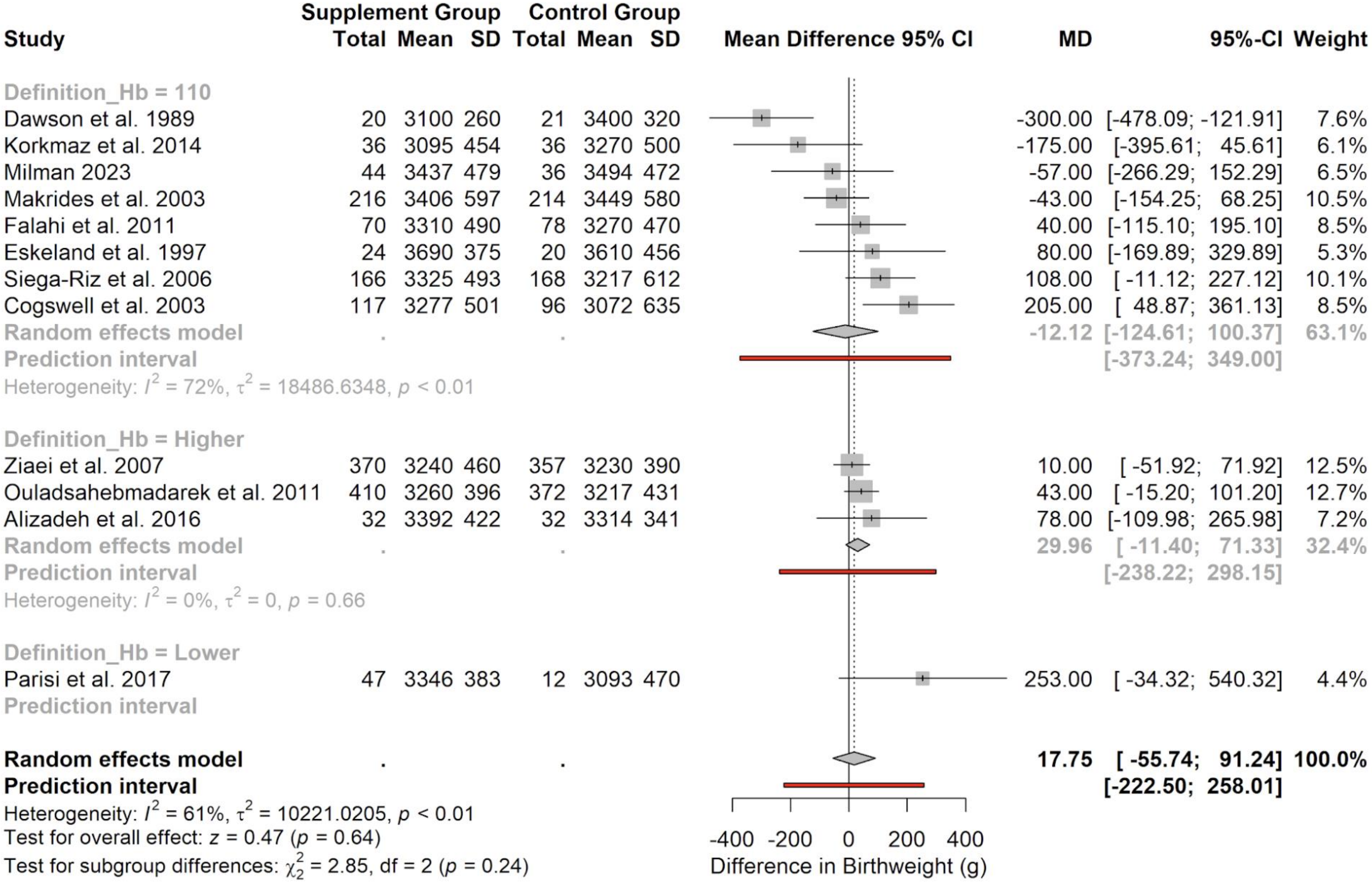

Figure S26 - Forest plot showing the effect of iron supplementation on birthweight with subgroup analysis based on when during pregnancy iron supplements were started.

	Supplement Group			Control Group					
**Study**	**Total**	**Mean**	**SD**	**Total**	**Mean**	**SD**	**MD**	**95%-CI**	**Weight**
**Start = First Trimester**									
Dawson et al. 1989	20	3100	260	21	3400	320	-300.00	[-478.09; -121.91]	7.6%
Korkmaz et al. 2014	36	3095	454	36	3270	500	-175.00	[-395.61; 45.61]	6.1%
Ziaei et al. 2007	370	3240	460	357	3230	390	10.00	[-51.92; 71.92]	12.5%
Ouladsahebmadarek et al. 2011	410	3260	396	372	3217	431	43.00	[-15.20; 101.20]	12.7%
Eskeland et al. 1997	24	3690	375	20	3610	456	80.00	[-169.89; 329.89]	5.3%
Parisi et al. 2017	47	3346	383	12	3093	470	253.00	[-34.32; 540.32]	4.4%
**Random effects model**	.			.			-23.96	[-161.87; 113.95]	48.7%
**Prediction interval**								[-475.24; 427.32]	
Heterogeneity: $I^2 = 73\%$, $\tau^2 = 21467.6062$, $p < 0.01$									
**Start = Second Trimester**									
Milman 2023	44	3437	479	36	3494	472	-57.00	[-266.29; 152.29]	6.5%
Makrides et al. 2003	216	3406	597	214	3449	580	-43.00	[-154.25; 68.25]	10.5%
Falahi et al. 2011	70	3310	490	78	3270	470	40.00	[-115.10; 195.10]	8.5%
Alizadeh et al. 2016	32	3392	422	32	3314	341	78.00	[-109.98; 265.98]	7.2%
Siega-Riz et al. 2006	166	3325	493	168	3217	612	108.00	[-11.12; 227.12]	10.1%
Cogswell et al. 2003	117	3277	501	96	3072	635	205.00	[48.87; 361.13]	8.5%
**Random effects model**	.			.			56.39	[-23.73; 136.51]	51.3%
**Prediction interval**								[-154.08; 266.86]	
Heterogeneity: $I^2 = 41\%$, $\tau^2 = 4075.3637$, $p = 0.13$									
**Random effects model**	.			.			**17.75**	**[-55.74; 91.24]**	**100.0%**
**Prediction interval**								**[-222.50; 258.01]**	

Heterogeneity: $I^2 = 61\%$, $\tau^2 = 10221.0205$, $p < 0.01$

Test for overall effect: $z = 0.47$ ($p = 0.64$)

Test for subgroup differences: $\chi^2_1 = 0.98$, df = 1 ($p = 0.32$)

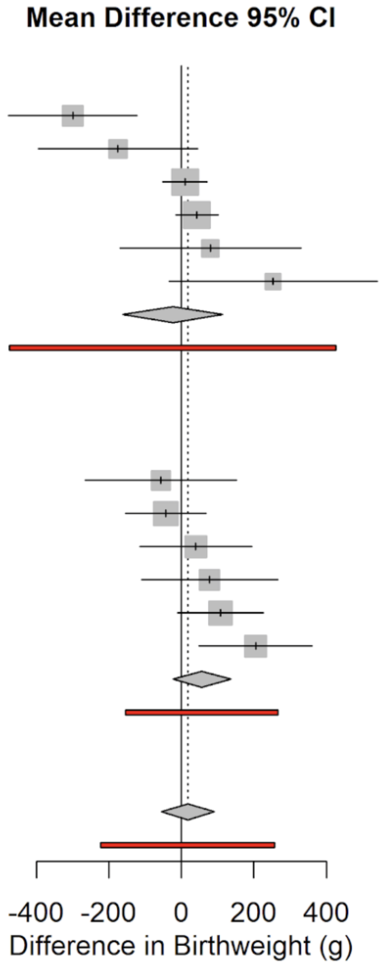

Figure S27 - Forest plot showing the effect of iron supplementation on birthweight with subgroup analysis based on human development index in the country and year of the study.

Study	Supplement Group Total	Supplement Group Mean	Supplement Group SD	Control Group Total	Control Group Mean	Control Group SD	MD	95%-CI	Weight
**HDI_class = Very High**									
Dawson et al. 1989	20	3100	260	21	3400	320	-300.00	[-478.09; -121.91]	7.6%
Korkmaz et al. 2014	36	3095	454	36	3270	500	-175.00	[-395.61; 45.61]	6.1%
Milman 2023	44	3437	479	36	3494	472	-57.00	[-266.29; 152.29]	6.5%
Makrides et al. 2003	216	3406	597	214	3449	580	-43.00	[-154.25; 68.25]	10.5%
Eskeland et al. 1997	24	3690	375	20	3610	456	80.00	[-169.89; 329.89]	5.3%
Siega-Riz et al. 2006	166	3325	493	168	3217	612	108.00	[-11.12; 227.12]	10.1%
Cogswell et al. 2003	117	3277	501	96	3072	635	205.00	[48.87; 361.13]	8.5%
Parisi et al. 2017	47	3346	383	12	3093	470	253.00	[-34.32; 540.32]	4.4%
**Random effects model**	.			.			**3.96**	**[-123.31; 131.22]**	**59.0%**
**Prediction interval**								**[-409.04; 416.95]**	
Heterogeneity: $I^2 = 74\%$, $\tau^2 = 24270.6575$, $p < 0.01$									
**HDI_class = High**									
Ziaei et al. 2007	370	3240	460	357	3230	390	10.00	[-51.92; 71.92]	12.5%
Falahi et al. 2011	70	3310	490	78	3270	470	40.00	[-115.10; 195.10]	8.5%
Ouladsahebmadarek et al. 2011	410	3260	396	372	3217	431	43.00	[-15.20; 101.20]	12.7%
Alizadeh et al. 2016	32	3392	422	32	3314	341	78.00	[-109.98; 265.98]	7.2%
**Random effects model**	.			.			**30.63**	**[-9.34; 70.60]**	**41.0%**
**Prediction interval**								**[-57.12; 118.38]**	
Heterogeneity: $I^2 = 0\%$, $\tau^2 = 0$, $p = 0.84$									
**Random effects model**	.			.			**17.75**	**[-55.74; 91.24]**	**100.0%**
**Prediction interval**								**[-222.50; 258.01]**	

Heterogeneity: $I^2 = 61\%$, $\tau^2 = 10221.0205$, $p < 0.01$

Test for overall effect: $z = 0.47$ ($p = 0.64$)

Test for subgroup differences: $\chi^2_1 = 0.15$, df = 1 ($p = 0.70$)

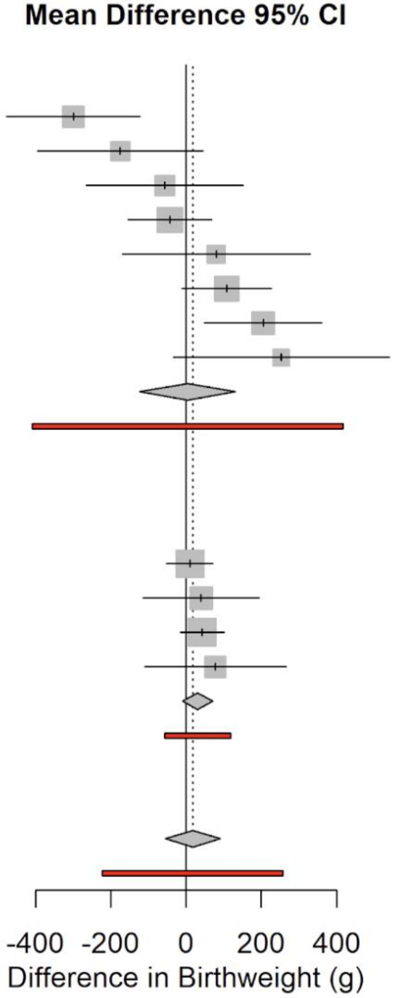

Figure S28 - Forest plot showing the effect of iron supplementation on rate of caesarean section.

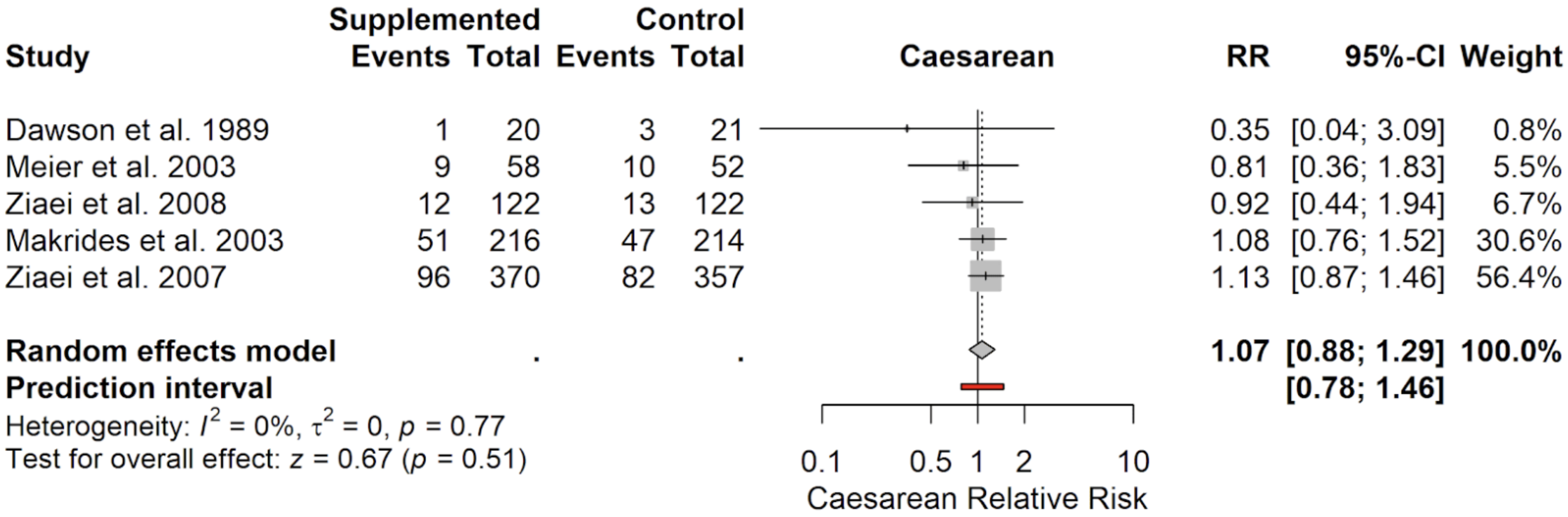

Figure S29 - Forest plot showing the effect of iron supplementation on rate of preterm births.

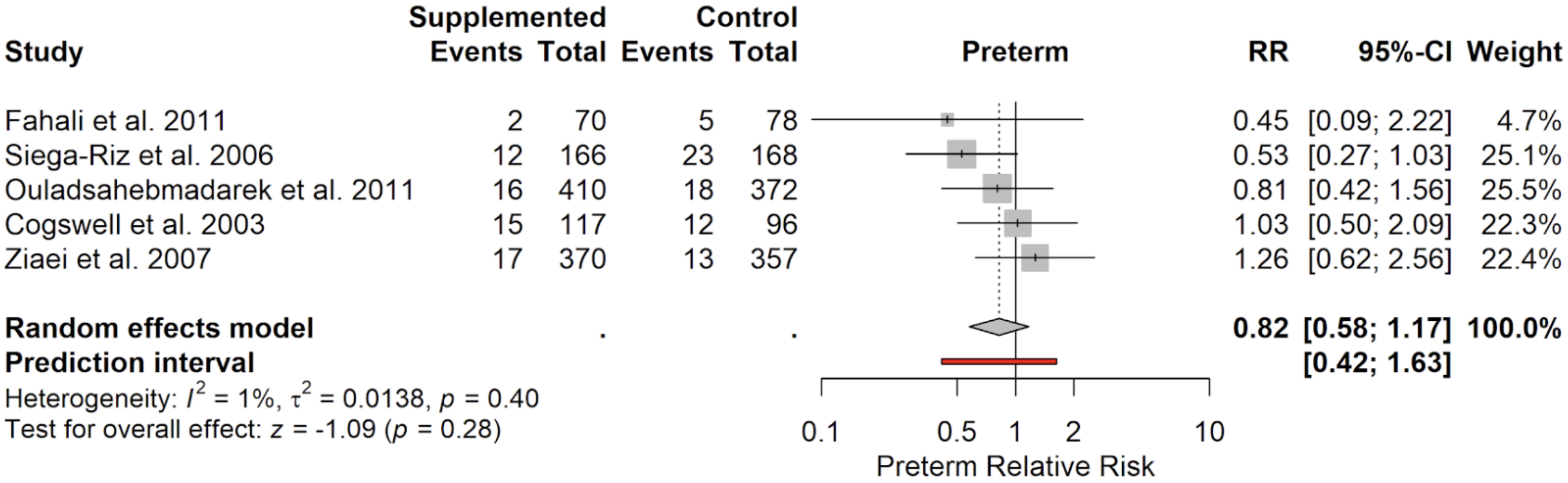

Table S30 - Table showing reporting of side effects in the included studies.

Study	Side effects reported	GI side effects			Other harms/side effects reported (comparison of significance)
		Intervention	Control	Comparison significance	
Svanberg et al. (1975)	No	-	-	-	-
Puolakka et al. (1980)	No	-	-	-	-
Palgi et al. (1981)	Yes	61/205	-	-	-
Buytaert et al. (1983)	No	-	-	-	-
Dawson et al. (1989)	Yes	-	-	-	Average gestational age (NI)<br>Serum Zinc concentration (NS)
Eskeland et al. (1997)	Yes	-	-	-	Women out of work (p<0.05; placebo > intervention)
Meier et al. (2003)	Yes	Nausea 35/58<br>Vomitting 21/58<br>Constipation 15/38<br>Diarrhea 8/38	Nausea 30/53<br>Vomitting 15/53<br>Constipation 12/53<br>Diarrhea 12/53	NS (Fisher's exact test)	Gestational age (NI)<br>Apgar scores (NI)
Cogswell et al. (2003)	Yes	-	-	-	Low birth weight (p=0.003; greater incidence in placebo)<br>Small for gestational age (p = 0.014; greater incidence in placebo group)<br>Birth length (NS)<br>Experience of any side effect (NS)
Makrides et al. (2003)	Yes	nausea 58/200<br>stomach pain 70/200<br>vomiting 24/200	nausea 54/193<br>stomach pain 57/193<br>vomiting 29/193	NS (Chi-square test)	Gestational age at birth (NS)<br>Serum Zinc at delivery (NS)<br>Birth Length (NS)<br>Neonatal death (NS)<br>Low Apgar score (NS)
Siega-Riz et al. (2006)	Yes	-	-	-	Incidence of low birthweight (NS)<br>Gestational age at delivery (NS)
Ziaei et al. (2007)	Yes	-	-	-	Hypertension disorder (p=0.05)<br>Small for gestational age (p=0.035; greater incidence in intervention)<br>Perinatal mortality rate (NS)
Harvey et al. (2007)	Yes	-	-	-	Zinc absorption (NS)
Ziaei et al. (2008)	No	-	-	-	-
Ozyigit et al. (2008)	Yes	-	-	-	Oral glucose load test (p=0.05)
Falahi et al. (2011)	Yes	-	-	-	Birth length (NS)<br>Low birthweight (<2500g) (NS)<br>Gestational age at delivery (NS)
Ouladsaheb madarek et al. (2011)	Yes	-	-	-	Pregnancy induced hypertension (p=0.04; lower incidence in placebo)<br>IUGR, PROM, preeclampsia oligihydramnious and placental abruption (all others NS)
Parisi et al. (2017)	No	-	-	-	Apgar score (NS)<br>Gestational weeks at delivery (NS)
Abioye et al. (2023)	Yes	-	-	-	Neonatal and perinatal mortality (NS)
Korkmaz et al. (2014)	Yes	-	-	-	Oligohydramnios (p<0.001; higher incidence in intervention)
Jafarbegloo et al. (2015)	Yes	*<br>nausea (2,15)<br>vomiting (0,3)<br>diarrhoea (0,0)<br>constipation (4,12)<br>loss of appetite (0,4)<br>heart burn (3,15)<br>abdo pain (0,2)	*<br>nausea (2,7)<br>vomiting (1,5)<br>diarrhoea (1,1<br>constipation (2,2)<br>loss of appetite (0,2)<br>heart burn (1,4)<br>abdo pain (1,0)	NS (Chi-square test)	-
Alizadeh et al. (2016)	No	-	-	-	-
Tholin et al. (1995)	Yes	-	-	-	Blood manganese level (NS)
Milman (2023)	Yes	-	-	-	Gestational diabetes (NS)
